# Under the influence of nature: The contribution of natural capital to tourism spend

**DOI:** 10.1371/journal.pone.0269790

**Published:** 2022-06-22

**Authors:** Alice Fitch, Jake Kuyer, Natalya Kharadi, Jacob Gower, Caroline Roberts, Nicola Dewey, Stephen Hull, Laurence Jones

**Affiliations:** 1 UK Centre for Ecology & Hydrology, Environment Centre Wales, Bangor, United Kingdom; 2 Economics for the Environment Consultancy, London, United Kingdom; 3 SQW, Oxford Centre for Innovation, Oxford, United Kingdom; 4 APBmer, Quayside Suite, Medina Chambers, Southampton, United Kingdom; 5 Liverpool Hope University, Liverpool, United Kingdom; Gebze Teknik Universitesi, TURKEY

## Abstract

Tourism and outdoor leisure is an important economic sector for many countries, and has a substantial reliance on natural capital. Natural capital may be the primary purpose for tourism, or it may be a secondary factor, where the choice of location for a leisure activity is influenced by natural capital. Typically, when valuing tourism and outdoor leisure, all expenditure associated with the activity is assigned to the ecosystem it occurs in. However, this value illustrates the *dependency* on natural capital, rather than the *contribution* of natural capital. In natural capital accounting, a major challenge is to separately identify the contribution of natural capital from that of other forms of capital. In this study we develop a transparent and repeatable method that is able to attribute the contribution of natural capital (here defined as ecosystems) to the output of multiple tourism and outdoor leisure activities. Using national statistics from Great Britain, we calculate the natural capital contribution to tourism spend by activity at a national and regional scale, and for a case study map and value the contributing ecosystems. We estimated that, out of a total £36 billion spent on tourism and leisure activities in 2017, £22.5 billion was attributable to natural capital. This equates to 0.9% of the UK GDP. The Gross Value Added component of this attributable was £10.5 billion, equivalent to 0.4% of the UK GDP. Regions with the highest natural capital contribution in Great Britain were Scotland and Wales, with the lowest being Greater London and the West Midlands in England. For the case study, the ecosystems with the greatest contribution to terrestrial activities were marine and enclosed farmland. These methods can be applied worldwide for anywhere with aggregate economic statistics on expenditure associated with tourism and outdoor leisure, with the aid of open source GIS datasets.

## Introduction

Tourism is a substantial component of the global economy, in 2019 tourism contributed approximately 10.4% to global GDP (US$ 9,170 billion) [1], and tourism is forecast to be one of the fastest growing sectors over the next decade [1]. In some countries it is the biggest single source of revenue; tourism contributed US$ 90.0 billion (22.5%) to total GDP in the Philippines, and US$ 175.6 billion (15%) for Mexico [1]. The appeal of a country or region for tourism is complex, consisting of many elements including its culture, history, climate, accessibility, and natural environments. In the tourism and outdoor leisure (T&OL) industry, much of the revenue generated is highly dependent on natural capital [2]. For example, bird watching in Białowieża Forest, Poland, is estimated to generate around US $2 million per year in expenditure [3], coral reefs generate US $35.8 billion dollars per year in expenditure for associated countries [4], and globally national parks are estimated to generate US $600 billion per year in direct in-country expenditure [5]. However, separating out the role of natural capital from the other contributing elements to tourism remains a major challenge.

**Fig 1:**
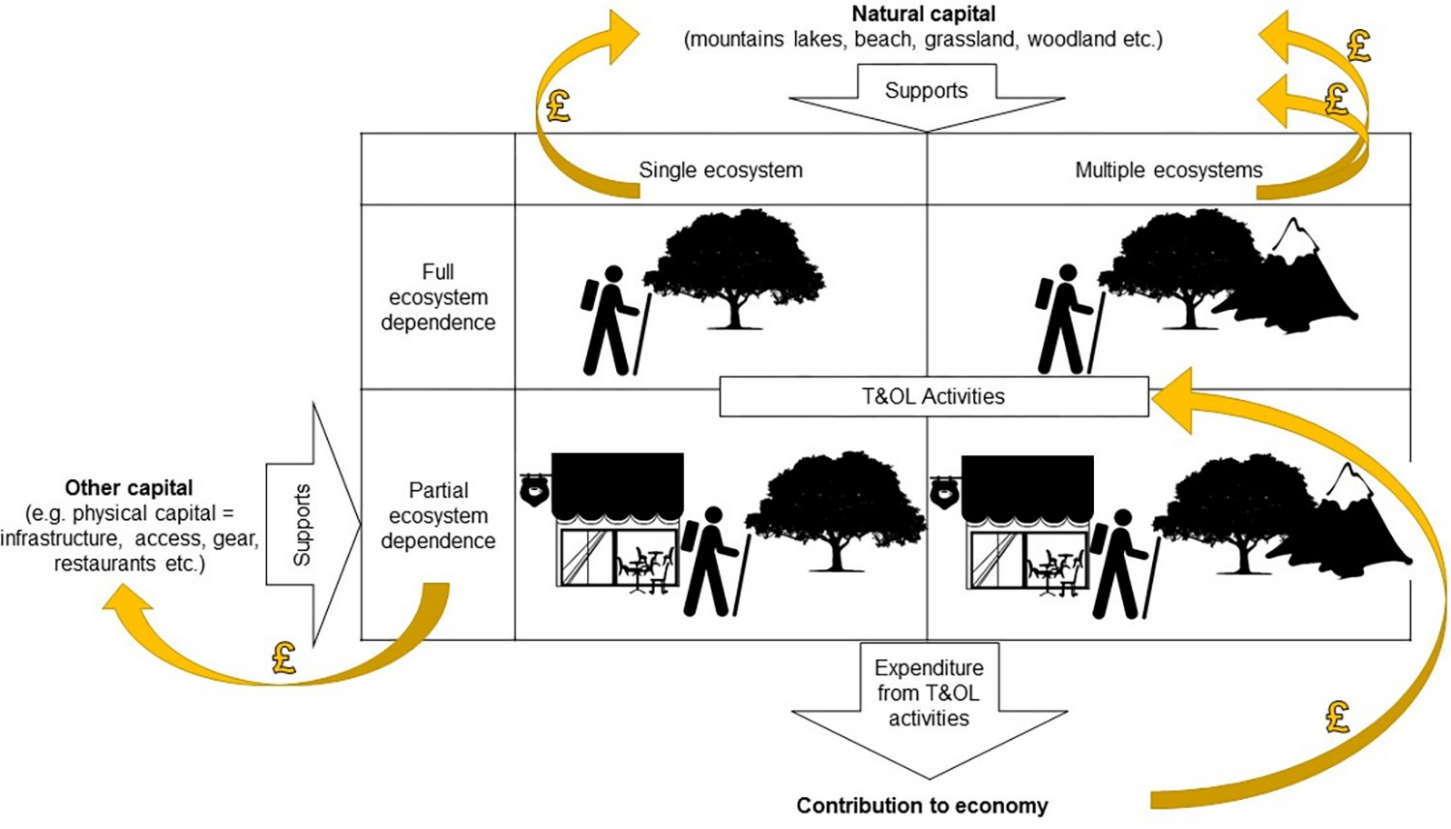
Contribution of other capital (such as physical) and natural capital to T&OL activities and consequently economy. Ecosystems and other capital support T&OL activities. T&OL activity expenditure is therefore is attributable to natural capital.

Here we simplify natural capital as ecosystems (woodland, heathland, grassland, coasts, etc.), but natural capital has much wider definitions encompassing specific biotic and abiotic components of ecosystems and the ecological processes and functions they support [6]. Natural capital contributes to tourism in a number of ways. It may be the primary purpose for tourism (nature-based tourism), for example visiting canyons, beaches, or to see specific flora and fauna. Alternatively, it may play a secondary role, where the primary purpose is an outdoor leisure activity, for instance to play golf or go for a bike ride, but the choice of location is influenced by natural capital: a golf course by the sea, or a country park for a bike ride. In almost every instance, natural capital is not the sole contributor to T&OL. The majority of tourism activities cannot occur (or would occur to a much lesser extent) without ‘other factors of production’ that support participation in the activity (Fig 1). Of these other factors (henceforth referred to as other capital), physical capital including built infrastructure, equipment, etc. is a key aspect, but see Jones et al [7] for a discussion of social and cultural capital which also play an important role in recreation activity. For instance, in order to go cycling you need a bike (activity specific equipment) and most cyclists need built paths or trails (infrastructure). The ways in which other capital facilitates the use of natural capital for T&OL are widely recognised. For example, Heagney et al. [8]) found that built infrastructure, particularly roads and car parks, increased the usage of national parks in New South Wales, Australia.

In this context, while all expenditure associated with a T&OL activity may be *dependent* on natural capital (i.e. the expenditure would not occur if the natural capital was not present), only a subset of this value should be attributed to the natural capital itself, as other factors of production are also involved (e.g. built infrastructure, equipment, labour). Therefore, to properly account for the natural capital contribution to T&OL, the associated expenditure must be attributed to each of the contributing factors. Failure to do so would risk double counting with value already accounted for elsewhere (e.g. national accounts as reflected in GDP). Although previous studies have improved our understanding of the value of natural capital to tourism, they have generally focused on a particular natural capital type associated with tourism, such as coral reefs [e.g. 4] or a defined tourism area such as national parks [e.g. 5, 9, 10]. Neither of these approaches seek to partial out the contribution from natural capital and other forms of capital. To summarise, current approaches to estimate the economic value of natural capital to tourism have mostly considered the *dependency* that T&OL has on natural capital, rather than the *contribution* of natural capital (amongst other types of capital) to T&OL. Quantifying the specific contribution of natural capital and other capital to T&OL is a major knowledge gap and is only starting to be addressed [11].

There are two ways to approach the task of attributing T&OL value to natural capital: ‘top-down’ or ‘bottom up’. The top down method typically starts with aggregate tourism expenditure data at a national level, often for only a single activity or ecosystem, and then breaks this down to identify all tourism spend that is dependent on a particular natural feature. Spalding et al. [4] applied this method to assess the global contribution of coral reefs to tourism, using social media data and GIS, and splitting this further into on-reef tourism and reef-adjacent tourism. The more common ‘bottom-up’ method uses expenditure or value per trip and visitation rates, often calculated using a random utility travel cost model to estimate and attribute leisure spend (see [8] for further detail on random utility travel cost modelling). Balmford et al. [5] applied this method, utilising visitation data and characteristics of protected areas to model visits globally to protected areas, producing a global estimate of tourism value (direct expenditure and consumer surplus) associated with protected areas.

In addition to calculating the contribution of natural capital, there is increasing interest in mapping which natural capital is contributing to T&OL, and where tourism activity is occurring. Tourism tends to aggregate in particular locations, forming hotspots [12]. At the Great Barrier Reef 80% of tourism takes place in 7% of the region [13] [p112]. Spatial mapping of tourism is vital for future planning and management of tourism activities [2]. For example, to minimise or manage conflict between different user groups [14], between visitors and residents [15, 16], with wildlife [17], as well as damage to natural environments [18]. All natural capital within a region has the *potential* to contribute to tourism, but visitors/users–identified by the spatial analysis of tourism activities or visitation rates–result in a *realised* contribution to tourism. Knowing the locations where activities happen can help in the task of identifying which natural capital is supporting that activity, and can help understand dependencies between natural capital types. For example, tourism value can be attributed to different natural capital types inside a national park by knowing where visitors spend their time [19]. When applying a top-down approach with national level tourism data, identifying the natural capital that is *actually* contributing to the activity is significantly harder, since only a high-level activity description and broad geographic region of where visits have occurred are known.

Tourism is a growth industry, and while there are many positive aspects to T&OL, its growth can place pressure on the underlying ecosystems that contribute to T&OL activities, causing degradation if not managed sustainably, and this in turn can reduce their viability as tourism assets [18, 20]. In this context, evidence (aggregated and spatially explicit) on the economic value of ecosystem assets to the tourism sector can support efficient management of the ecosystems which underpin T&OL, and help to make the case for investment in maintaining and enhancing these valuable ecosystem assets.

In this paper, we outline an approach to calculate the amount of tourism expenditure that can be attributed to natural capital (specifically ecosystems) versus other capital, and how this natural capital can be identified to allow spatial disaggregation of value between different ecosystems. We use Great Britain (GB) as an example, making use of national T&OL expenditure data where the precise locations and ecosystems contributing are unknown. For each T&OL activity we: 1) Calculate the value attributable to natural capital (ecosystems) rather than other capital, and 2) For a case study area in South Wales, we determine spatially which natural capital (ecosystems) are contributing to each activity, and use that data to apportion the natural capital value to each ecosystem. In doing so, we address some of the challenges of developing a practical approach making use of existing data, which combines national-level expenditure-based economic data and a more spatially refined GIS analysis approach. We conclude by identifying aspects of the approach which would benefit from further refinement.

## Method

The basis of the approach is that the contribution of natural capital to economic activity is reflected in the market prices paid by people engaging in activities that are enabled or enhanced by nature. In line with current guidance on natural capital accounting [21], this approach considers only the exchange value of the contribution from natural capital. The welfare value that is created additional to the market mechanism is not represented in the results. Natural capital acts like a factor of production of economic value through supporting T&OL activities. To assess the contribution of natural capital to the market value of these activities, it must be split out from other factors of production.

**Fig 2:**
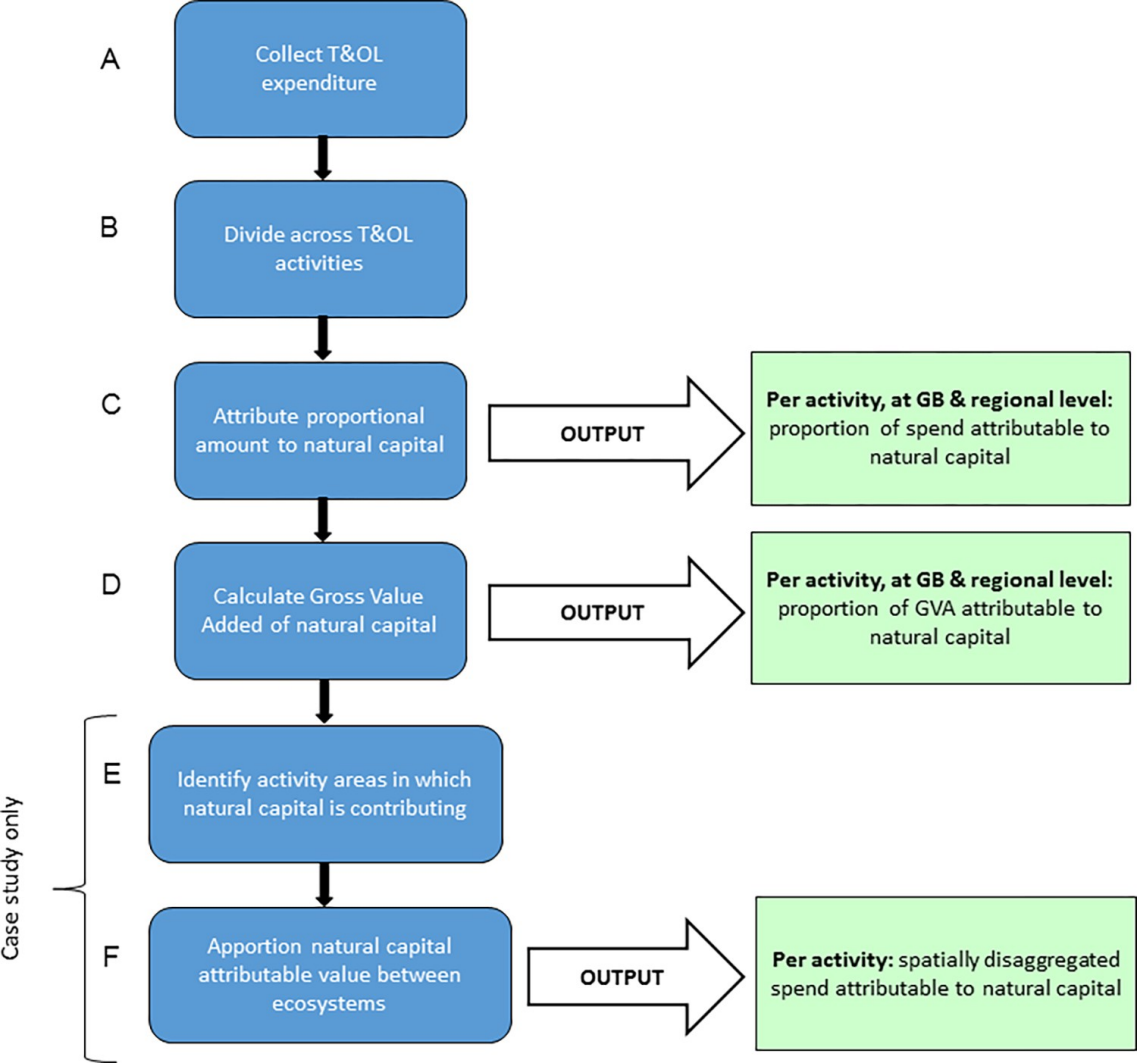
Flow diagram of method. Stages for calculating natural capital contribution to T&OL expenditure.

The schema in Fig 1 illustrates the different components contributing to T&OL spend, and their inter-relations. In almost all instances, both natural capital and other capital act in combination to enable T&OL. The process of calculating the contribution of natural capital to tourism spend is broken down in six stages, outlined in the workflow in Fig 2. A worked example is provided in supplementary information (S1 File).

### Collect tourism and outdoor leisure expenditure (A)

Data on the expenditure made by T&OL visitors, as well as the activities they undertook, was obtained at both national and regional level (NUTS-1: Wales, Scotland, plus nine statistical regions in England) from three main surveys, for the year 2017:

- Great Britain Day Visitor Survey (GBDVS) (for trips of more than 3 hours) [22, 23]
- Great Britain Tourism Survey (GBTS) (for domestic, overnight visitors) [24]
- International Passenger Survey (IPS) which covers international visitors [25, 26]

All three surveys are undertaken annually and cover GB, providing estimates of total expenditure and activities undertaken on trips. The questions used to determine the types of tourism and leisure activities differ between surveys and can vary year to year within surveys. However, despite these differences, collectively these three surveys provide the most reliable expenditure estimates of the main sources of T&OL spend at a national level.

### Divide across tourism and outdoor leisure activities (B)

The relative contribution of natural capital and other capital to T&OL is activity-specific. The categories and descriptions of activities across the three surveys were harmonised using activity typologies based on TNS [27]. Twenty four T&OL activities were identified, 16 of which stem from the GBTS with an additional eight activities identified through the GBDVS detailed activity list [23]. The resulting activity list is provided in supplementary information (S2 File).

#### Expenditure attributable to T&OL activities–national level

At the national level, expenditure was attributed to each of the 24 activities using an approach based on the research of the three National Tourist Boards (VisitEngland, VisitScotland, and VisitWales) and TNS [27]. The approach assessed the degree of importance of the activity for taking a trip (i.e. motivation), and how much of the trip expenditure was directly related to participation in that activity along with other activities undertaken on the same trip in order to obtain the expenditure attributable to natural capital. “Attributable expenditure” is defined as “*an estimate of the amount of money spent on day or overnight visits which were motivated by being able to participate in a* ***specific activity*** *when the decision was made to take the trip*” [27] [p5]. It includes money spent on accommodation, food and drink, and transport, as well as any **direct costs** associated with engaging in the activity. The attribution method devised is applicable at the **national** level, and aims to deal with the common situation in which multiple activities are identified as motivating the trip:

1. Measure the expenditure associated with trips including the activity
2. Estimate the extent to which individual activities are the reason for taking a trip
3. Identify the average number of other activities undertaken on trips
4. Use data from steps 2 and 3 (if applicable) to calculate the percentage share of expenditure attributable to each activity

Following these steps, we estimated the total attributable expenditure across the 24 T&OL activities, for each survey. Steps 2 and 3 were applied to the GBDVS and GBTS using 2015 omnibus survey results [27]. Steps 2 and 3 could not be applied to the IPS, as the IPS data that is publicly accessible does not provide detail on activities undertaken by visitors, only high-level estimates on total expenditure [26]. Omnibus survey results were not applied to IPS due to potentially different motivations between international and domestic tourists (omnibus survey involved GB residents only). Therefore, to achieve step 4 the percentage attributable share for each activity was taken directly from TNS [27]

#### Expenditure attributable to T&OL activities–regional level

At the regional level, expenditure was attributed to each of the 24 activities using the percentage share attributable derived at the national level in step 4. Steps 2 and 3 could not be applied to GBDVS and GBTS regional breakdown as research into activity motivations at the regional level have not been undertaken. Applying the share of spend attributable to each activity at the national level assumes that activity motivations observed at the national level are representative of the regional level, and there is no variation in activity expenditure between regions. Regional T&OL expenditure is based on GBDVS and GBTS only, IPS data was excluded from this part of the analysis as the IPS data that is publicly accessible does not provide information on where overseas visitors travelled to within the UK [26].

### Attribute proportional amount of activity expenditure to natural capital (C)

Having identified the expenditure associated with each T&OL activity, the next step is to attribute a portion to the contribution from natural capital. We derived this by evaluating the degree to which an activity is motivated by, or dependent on, the natural environment compared to other capital. We refer to this as ‘ecosystem contribution’.

A simple scoring system was developed, drawing on descriptions in Barton, Obst [28], to apportion value between natural and other capital for each activity based on how an activity relates to the natural environment. A five-point percentage scale was constructed with associated descriptions of an activities’ relationship with natural capital (Table 1).

**Table 1:** Ecosystem attribution scoring table.

% ecosystem contribution	Descriptor
100%	Natural capital essential to experience
75%	Nature capital greatly improves experience
50%	Nature capital moderately improves experience
25%	Nature capital secondary to experience
0%	Natural capital inconsequential to experience

The ecosystem contribution of an activity may vary due to the location where the activity takes place. Therefore, three broad activity location categories were adopted from GBDVS [22, 23] and used when deriving the ‘ecosystem contribution’.:

- Built-up (city/large town; small town)
- Rural (village; rural countryside)
- Coastal (seaside resort or town; seaside coastline–a beach; other seaside coastline)

A ‘location contribution’ percentage was derived using the breakdown of visits from GBDVS between these three activity locations [22, 23]. The ‘location contribution’ percentages for each activity are provided in S1 Table.

To obtain the initial percentages in Table 1 for each activity in each location category, two framing questions were used to separate the importance of natural capital and other capital. Other capital in this context refers to ‘other factors of production’ that support participation of the activity, of which physical capital is likely to be the main aspect:

1. Would the activity occur without the natural environment?
2. Would the activity occur without other capital (e.g. built infrastructure)?

Based on a response of ‘yes’, ‘no’, or ‘partially’, a ‘baseline ecosystem contribution’ percentage was obtained from Table 2 for each of the 24 activities, for each location category. Final percentage of expenditure attributable to national capital was the sum product of the ‘location contribution’ and ‘baseline ecosystem contribution’.

**Table 2:** Attribution rates between ecosystem contribution and other capital.

		*Would the activity occur without natural capital*?		
		YES	PARTIAL	NO
*Would the activity occur without other capital*?	**YES**	NC = 50%	NC = 75%	NC = 100%
		OC = 50%	OC = 25%	OC = 0%
	**PARTIA**L	NC = 25%	NC = 50%	NC = 75%
		OC = 75%	OC = 50%	OC = 25%
	**NO**	NC = 0%	NC = 25%	NC = 50%
		OC = 100%	OC = 75%	OC = 50%

Final ecosystem contribution was the sum product of ‘location contribution’ multiplied by ‘baseline ecosystem contribution’.

### Calculate GVA of natural capital (D)

In addition to working out expenditure attributable to natural capital for each activity, gross value added (GVA) was obtained. GVA was calculated by linking survey expenditure categories to the 2016 UK-Tourism Satellite Account (TSA) [29] tourism industries and using the GVA-to-output ratio from this UK-TSA. The GVA-to-output ratio for the UK tourism sector is calculated from the estimated total output and the estimated total gross value added of tourism industries and other industries ([29], Table 5). An additional tourism industry listed as ‘Other’ was created to allow inclusion of expenditure items from the GBDVS and GBTS surveys that are classed as ‘other consumption products’ or which may overlap with tourism industries (e.g. ‘special shopping’ or ‘entertainment’). GVA figures for each survey are estimated as the product of the UK-TSA GVA-to-output ratio and the GBDVS expenditure item proportions.

Each domestic survey provides data on expenditure items for GB as a whole, and separately for England, Scotland and Wales. At the GB level GBTS and IPS are treated the same, assuming GBTS expenditure distribution by expenditure category is representative of overseas tourists as well. When broken down to the devolved nations, this assumption is dropped as expenditure estimates do not include IPS data. For the nine NUTS-1 statistical regions of England, expenditure proportions for England (from GBDVS and GBTS) are applied, assuming there is no variation in expenditure on different items across the country. The GVA proportions are further broken down to account for each survey’s attributable expenditure to each activity, by multiplying the survey’s GVA proportion by the proportion of total attributable expenditure.

### Spatial allocation case study

To demonstrate the approach of identifying different ecosystems and spatially attributing the ecosystem contribution to tourism, we selected the county of Pembrokeshire in South Wales as a case study for 16 of the 24 activities. The county of Pembrokeshire (1,666 km^2^) was chosen as it is a popular tourism destination and contains a diversity of ecosystems. These include a highly scenic coastline which is a major draw for tourism, as well as inland cultural elements. This allowed testing of the terrestrial and marine activity disaggregation methods.

In order to obtain an expenditure value for Pembrokeshire, the value for each activity was estimated as a proportion of the national value for Wales based on GBDVS data. Using this method, Pembrokeshire was calculated to contribute 5.01% of the total value for Wales (3 year average (2015–2017) of GBDVS [22]).

### Identify activity areas in which natural capital is contributing (E)

**Fig 3:**
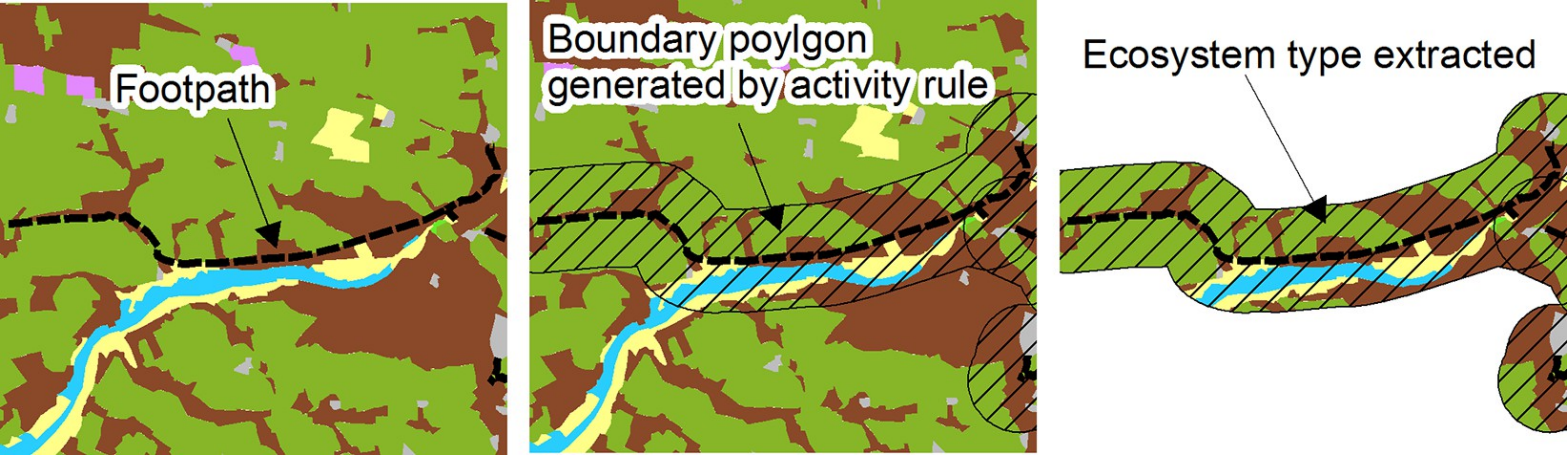
Extracting ecosystem type. Panels from left to right detail how the ecosystems contributing to activity were identified and their area extracted.

For each activity, we undertook mapping of the ecosystems that contribute in some way to that activity, or the experience while taking part in that activity in a location. This involved the creation of a set of specific rule bases for each activity. The activity specific rules applied, along with caveats, are provided in S2 Table. Spatial information was sourced that provided evidence for the likelihood of an activity to take place. This included ecosystems needed for the activity (e.g. beaches), local scale features (other capital) (such as footpaths, golf courses, Royal Society for the Protection of Birds reserves), and larger scale defined areas to which certain activity assumptions could be applied (such as National Parks, Areas of Outstanding Natural Beauty). Applying the activity specific GIS rules generated a boundary polygon within which natural capital was assumed to be contributing to the activity (Fig 3).

#### Apportion natural capital attributable value between ecosystems (F)

Using the generated boundary polygons, the type and area of different ecosystems was extracted. For terrestrial activities, habitat was extracted from the UK Land Cover Map [30]. Separately, the area of land with sea views [31] was calculated in order to attribute value for the marine component which occurs on land (termed ‘marine by proxy’). For attribution to terrestrial ecosystem types a two-stage process was followed. For activities with an essential dependency, such as fishing which is dependent on the presence of water, 80% of the value was apportioned to the essential ecosystems. For ecosystems which play a lesser role, but which may still influence the location where activities take place, the remaining 20% of the value was apportioned among them. Apportioning the value followed a two-factor weighting procedure, with one weight corresponding to the area of the ecosystem extracted for each activity, and a second weight corresponding to people’s preferences for particular ecosystem types in the landscape (derived from Swetnam, Harrison-Curran [32]). A worked example for ‘played golf’ is provided in S1 File. For the marine activities, spend was disaggregated based on ecosystem-activity zones. For ‘visiting a beach’, beaches with car parks less than 100m away were assigned 60% of the value, greater than 100m away assigned 30% of the value, and with no car parks nearby assigned 10% of the value. For ‘watersports’ the categories boardsports and vessel-based sports were created, with expenditure attributed equally between them. For ‘fishing-sea angling’ the categories shore-based angling and boat-based angling were created. Although more people are assumed to undertake shore angling (as it can be undertaken from any coastal area), people spend more money on boat-based angling trips, therefore expenditure was attributed equally between them. Fishing was split into terrestrial and marine in section B. The relevant attribution methods described above were then followed. The ‘marine by proxy’ category was calculated for terrestrial habitats, and then allocated to marine expenditure.

While the assumptions vary with each activity, a number of general principles are applied in this case study: Data layers are assumed to reflect current status. It is accepted that not all activity locations will have been captured in the process, however, that the output is representative of the most likely locations where activities are occurring. Wherever possible, datasets were used which are available (or a similar alternative) at a national scale to allow transferability of the method to other regions of the UK.

## Results

### Natural capital contribution to tourism spend

In 2017, T&OL activity expenditure in GB was £36 billion annually (Table 3), of which £22.5 billion is attributable to natural capital. When compared to the UK economy as a whole in 2017 (£2.6 trillion [33]), T&OL activity expenditure accounts for 1.4% of UK GDP, of which 0.9% comprises the contribution from natural capital. The contribution of natural capital to GVA through T&OL activities is £10.5 billion (out of the £22.5 billion), equivalent to 0.4% of the UK GDP.

**Table 3:** Tourism expenditure. Total tourism expenditure from each survey and the amount attributable to T&OL activities.

	*GBDVS (£ billions)*	*GBTS (£ billions)*	*IPS (£ billions)*	*Combined (£ billions)*
*Total tourism expenditure (£ billions)*	23.7	24.5	88	136.2
*Total expenditure attributable to T&OL activities (£billions)*	30	4.6	1.5	36.1

**Fig 4:**
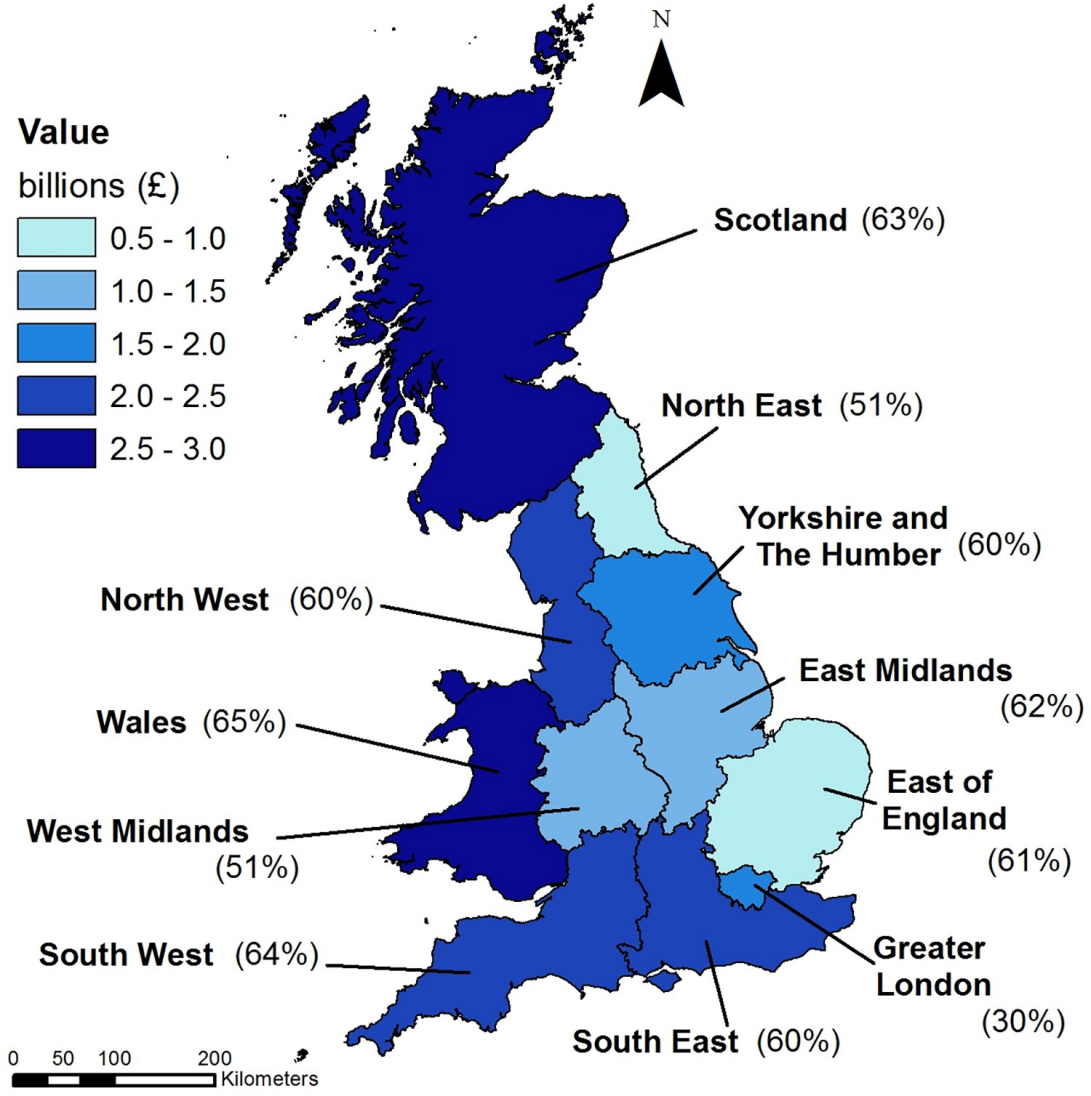
Natural capital contribution to T&OL expenditure broken down by region. T&OL expenditure attributable to natural capital, with percentage of total provided in brackets. Includes boundary of NUTS1 regions. Source: Office for National Statistics licenced under the Open Government Licence v3.0. Contains OS data © Crown copyright and database right 2019.

T&OL activity expenditure and amount attributable to natural capital per region is provided in Table 4. London has the lowest ecosystem attribution, with natural capital contributing only 30% to tourism spend, followed by the West Midlands and North East England at 51%. Wales has the highest ecosystem contribution at 65%, followed by South West England and Scotland at 64% and 63% respectively (Fig 4). These findings result from the difference in activities undertaken in each region (i.e. one does not go to the West Midlands to visit a beach). Note that the aggregated regional values do not match the national total, since these were based solely on expenditure data from domestic surveys (GBDVS and GBTS, excluding IPS).

**Table 4:** T&OL expenditure per region. Total activity expenditure, attribution to natural capital, and natural capital GVA for each region.

Region	Activity expenditure (£ millions)	Attribution to natural capital (£ millions)	GVA from natural capital (£ millions)
Scotland	4,014	2,534	1,169
Wales	4,210	2,750	1,278
North East England	1,020	524	244
North West England	3,369	2,021	942
Yorkshire and The Humber	3,025	1,810	844
East Midlands	2,009	1,239	577
West Midlands	2,215	1,120	522
East of England	1,432	873	407
London	5,857	1,776	828
South East England	3,579	2,156	1,005
South West England	3,415	2,200	1,025

### Natural capital contribution to national tourism spend, by activity

**Fig 5:**
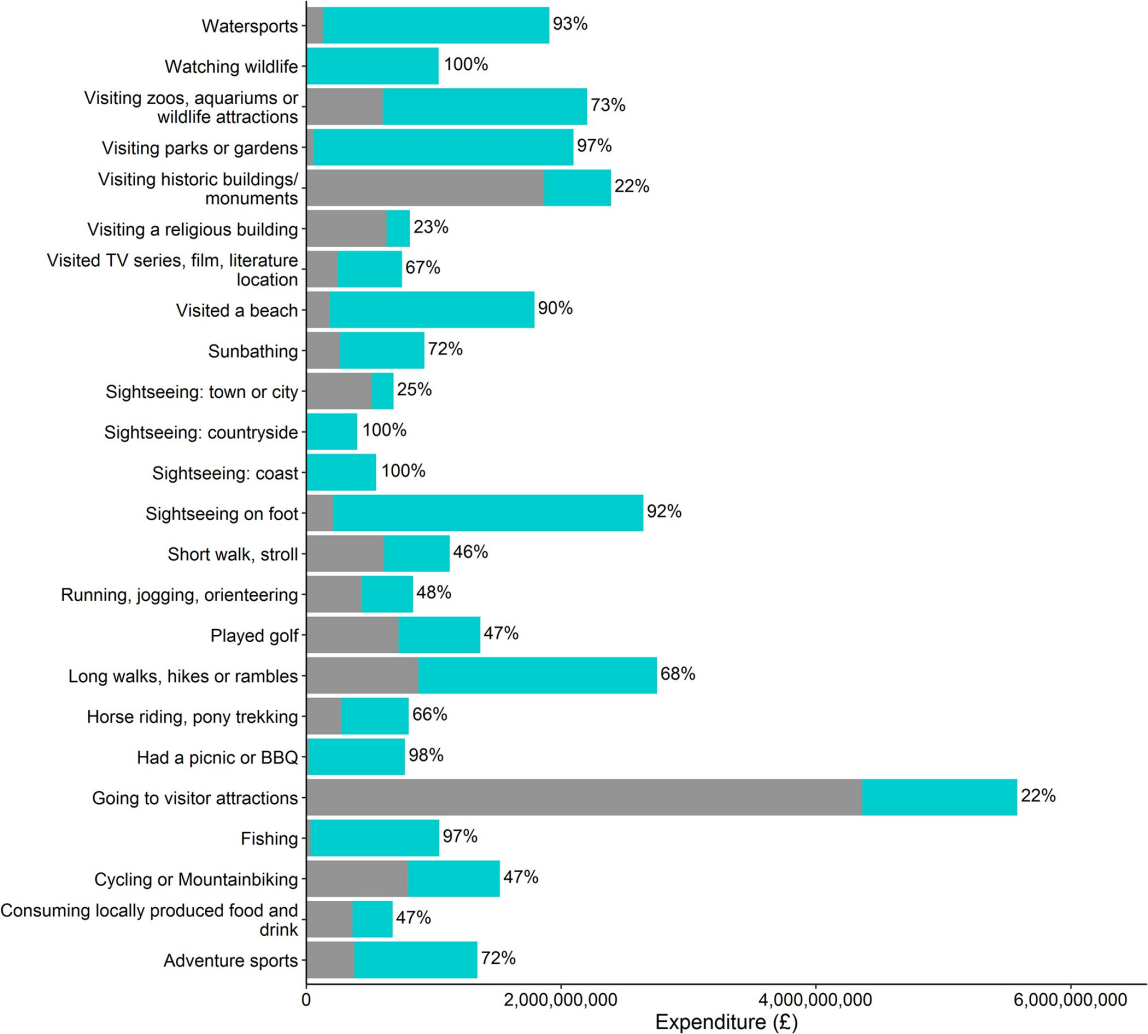
Natural capital contribution per activity. Graph showing natural capital (blue) and other capital (grey) contribution to each activity. The % natural capital contribution is shown at the end of each bar. Activity descriptions have been shortened to enable graph readability.

Natural capital contribution per activity is shown in Fig 5. For the activities “watching wildlife, bird watching, other nature”, “sightseeing/exploring at the coast”, and “sightseeing/exploring at the countryside”; 100% of the spend is attributable to natural capital. These are activities where natural capital is considered essential to the activity, and can in principle be undertaken without any other capital, although it may be facilitated by some types of infrastructure. At the other end of the scale “going to visitor attractions” and “visiting a historic building or monument”; natural capital makes relatively little contribution to the activity with only 22% of spend attributable to natural capital. The contribution is not deemed to be 0% as these features attract visitors in part because of their location (e.g. lighthouses on rocky coastlines, and landscaped grounds around stately homes).

The need for specific equipment to undertake the T&OL activity can be seen in the expenditure allocation for examples such as “fishing” and “watersports”, where the expenditure attributable to natural capital is not 100%, rather 97% and 93% respectively. In these examples, the ecosystem contribution is high because without the fish or the water, these activities could not exist. Whereas in the case of “played golf” and “cycling”, the ecosystem contribution is lower than other capital as natural capital is not essential to the activity.

### Spatial allocation case study–Pembrokeshire, South Wales

T&OL activity expenditure for Pembrokeshire was £211 million, £138 million of which is attributable to natural capital.

**Fig 6:**
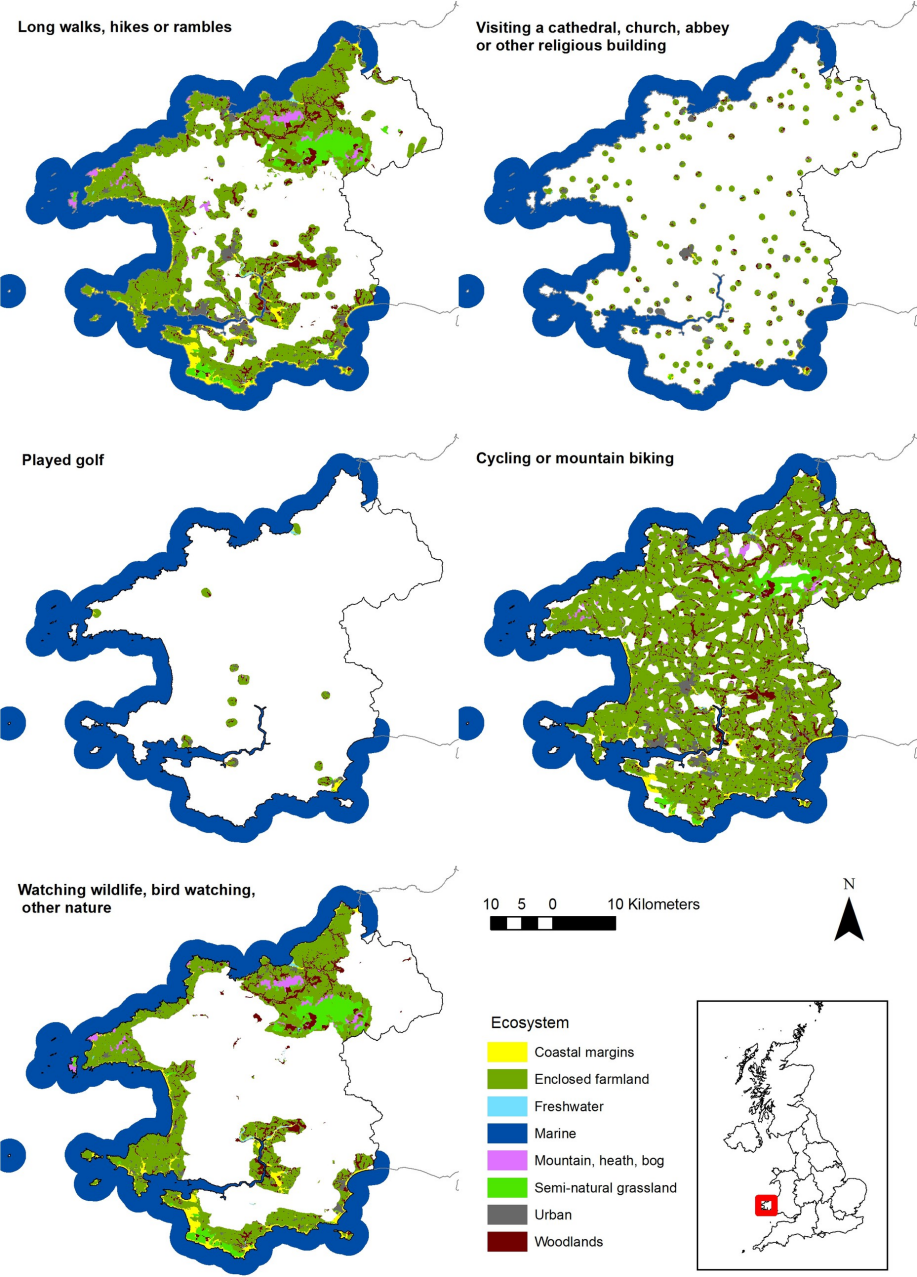
Location and type of ecosystems contributing to terrestrial activities. Includes boundary of Pembrokeshire and NUTS1 regions. Source: Office for National Statistics licenced under the Open Government Licence v3.0. Contains OS data © Crown copyright and database right 2019.

**Fig 7:**
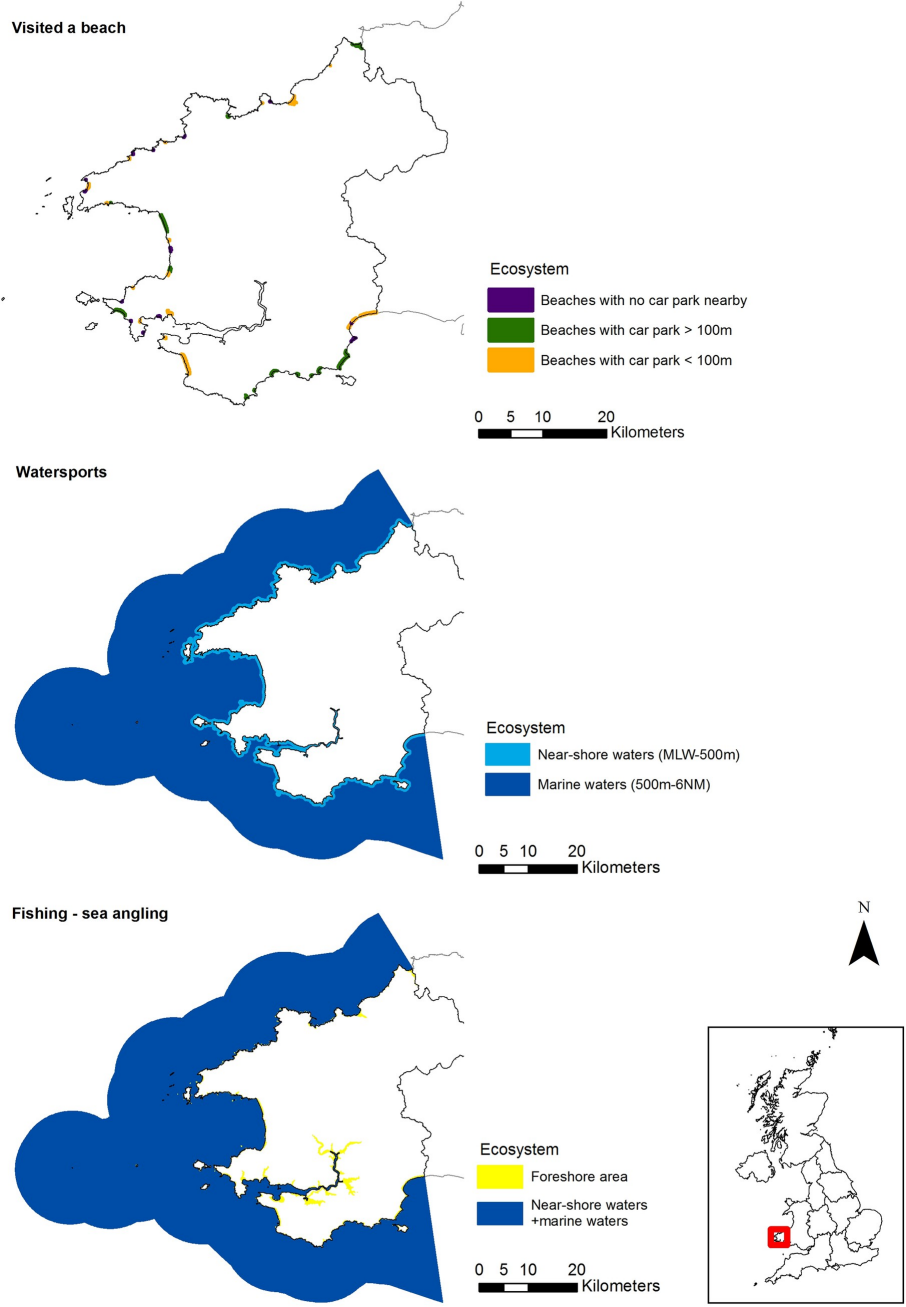
Location and type of ecosystems contributing to marine activities. Includes boundary of Pembrokeshire and NUTS1 regions. Source: Office for National Statistics licenced under the Open Government Licence v3.0. Contains OS data © Crown copyright and database right 2019.

For the 16 activities (£111 million attributable to natural capital), maps showing where the activity is most likely occurring and the location and type of ecosystem contributing are displayed in Fig 6 for five terrestrial examples (remaining supplied in S1 Fig), and in Fig 7 for marine activities. Maps for fishing reflect the split into sea angling and freshwater coarse and game fishing. The disaggregation of the expenditure attributable to natural capital between the different ecosystems identified through this activity mapping is displayed in Table 5 for terrestrial activities, and in Table 6 for the marine activities.

**Table 5:** Ecosystem breakdown of expenditure attributable to natural capital for 14 terrestrial activities in Pembrokeshire (£ thousands).

	Ecosystem								
Activity	Coastal margins	Enclosed farmland	Freshwater	Mountain, heath, bog	Semi-natural grassland	Urban	Woodlands	Total (terrestrial)	*Marine by proxy*
Visiting parks & gardens	20	2,111	1,973	-	-	599	2,169	6,872	*1*,*344*
Sightseeing in the countryside	48	4,835	26	142	400	-	661	6,112	*1*,*468*
Visiting zoos, aquariums or wildlife attractions	192	3,534	12	210	-	1,460	97	5,505	*2*,*618*
Watching wildlife, bird watching, other nature	1,124	4,289	46	210	568	-	643	6,880	*4*,*636*
Long walks, hikes or rambles	871	3,808	34	298	379	348	1,098	6,836	*7*,*417*
Going to visitor attractions	568	1,490	294	17	-	789	404	3,562	*1*,*307*
Visiting a cathedral, church, abbey or other religious building	55	602	9	3	5	151	76	901	*405*
Played golf	92	330	10	3	7	72	38	552	*323*
Eating and drinking locally produced food and drink	102	778	10	13	7	83	89	1,082	*544*
Visiting historic buildings or monuments	703	1,039	20	-	-	601	220	2,583	*791*
Visited a location associated with a TV series, film or literature	2,666	956	-	-	73	114	-	3,809	*2*,*922*
Sightseeing at the coast	4,016	2,026	108	207	133	325	278	7,093	*8*,*296*
Cycling or mountain biking	44	1,477	5	37	45	95	326	2,029	*903*
Fishing (freshwater angling)	-	531	2,844	2	11	46	121	3,555	*-*
**Total**	10,501	27,807	5,391	1,143	1,627	4,681	6,223	**57,373**	*32*,*975*

**Table 6:** Ecosystem breakdown of expenditure attributable to natural capital for 3 marine activities in Pembrokeshire (£ thousands).

Activity	Ecosystem							
	Foreshore	Near shore waters *MLW - 500m offshore*	Marine waters *500m offshore -6 NM*	Near shore waters + marine waters	Beach with car park < 100m	Beach with car park > 100m	Beaches with no car park nearby	Total
Fishing (sea angling)	1,397			1,397	-	-	-	2,793
Visiting a beach	-	-	-	-	4,920	2,493	861	8,274
Watersports	-	4,822	4,822	-	-	-	-	9,644
Expenditure by proxy from terrestrial								32,975
**Total value to marine**								**57,241**

“Cycling or mountain biking” (47% ecosystem contribution) has the potential to occur across Pembrokeshire due to the availability of suitable roads and tracks, as such enclosed farmland has the largest contribution to this activity (50%) (Fig 6, Table 5). Whereas, for “long walks, hikes, or rambles” (68% ecosystem contribution) the spatial location of suitable footpaths results in marine having the largest contribution to the activity (52%) (Fig 6, Table 5). Comparing “visiting a cathedral, church, abbey or other religious building” (23% ecosystem contribution) to “played golf” (47% ecosystem contribution), where golf courses are situated has resulted in a greater contribution of the coastal margins ecosystem to this activity (11% compared to 4%) (Fig 6, Table 5). For “watching wildlife, bird watching, other nature” (100% ecosystem contribution) ecosystems centred on the coastline and within protected/designated areas are contributing opposed to inland areas (Fig 6). For marine activities, T&OL expenditure is distributed equally across ecosystems regardless of ecosystem size, other than “visiting a beach” (90% ecosystem contribution) (Fig 7, Table 6). For this activity, more accessible beaches contribute the most to expenditure, and are spread across Pembrokeshire (Fig 7, Table 6).

The coastline is a major tourism driver for Pembrokeshire. For example, the Pembrokeshire coastal path is a key attraction for walkers and the coastline is home to an abundance of wildlife. This is reflected in marine having a high contribution value across all activities, and being the highest contributor of wildlife based activities (Table 5). Out of the activity total of £111 million, £54 million is attributable to the marine ecosystem (48%). Enclosed farmland is widespread across the region, and due to its presence as the dominant ecosystem (covering 76% of total area) results in a large contribution to all terrestrial activities (Table 5, Fig 6). Across the terrestrial activities, mountain moors and heath, and grasslands contribute the lowest value in this case study, mainly reflecting the relatively small area of these ecosystem types in Pembrokeshire (1% and 2% respectively).

## Discussion

This study calculates for the first time the contribution of natural capital to T&OL in GB, and provides a valuation of the total and the proportional ecosystem contribution per activity. Using a case study we demonstrate that it is possible to map the contribution of natural capital at fine resolution to show the spatial pattern of how each ecosystem type contributes to tourism spend for different activities. The need for increased sustainability of the tourism industry, often to protect the assets it indirectly or directly relies upon, is a well-recognised issue [34]. The results presented here provide an evidence base to feed into such policy and planning decision-making, and allows for more effective communication on this topic by enabling the comparison of the value of natural capital to tourism in the same terms that economic impact is often discussed.

The type of ecosystem contributing depends on the relationship of each activity with natural capital and spatial location within the UK. For “long walks, hikes or rambles” in Pembrokeshire the marine ecosystem was valued as the greater contributor, followed by enclosed farmland, due to the prominence of the Wales Coastal Path and its setting as part of a predominantly lowland agricultural landscape adjoining the sea. Mapping this activity in Scotland, for example, might produce a different balance of contributing ecosystems where mountain, heath and bog play a large role in the Scottish landscape. In another UK study Sen et al. [35] found that recreational visits to other ecosystems increased compared with a baseline of enclosed farmland. By contrast, Weyland and Laterra [36] did not find crop area to negatively affect recreation potential in Argentina, although this may be linked to the type of agriculture (e.g. homogenous vs heterogeneous [37]), or coincidental effects of farmland being the nearest suitable location in which to camp to access higher value natural capital. Enclosed farmland is clearly contributing to T&OL activities in Pembrokeshire; the majority of paths and trails for “long walks, hikes or rambles” and other T&OL activities in Pembrokeshire are located within or alongside enclosed farmland. Yet, whether the high valuation is due solely to the large coverage of this ecosystem type or because people choose open agricultural landscapes for undertaking that activity would require further investigation.

With few similar studies, it is difficult to find suitable comparators. Sen et al. [35] calculated per person per trip value for different types of natural capital, producing a country level value of natural capital supported activity but for domestic recreation. Their study utilised different socio-economic data and applied different assumptions. There is some discussion about whether such attribution between natural capital and other capital should occur. The argument against is the fact that the natural capital underpins the entirety of the value of the T&OL activity, since without the natural capital the T&OL activity would not be possible at all. While there is some merit to this argument, and it would simplify the assessment, in order to develop proxy-exchange values for the purposes of national accounting, the separate role of natural capital must be considered further. The corollary of assuming that all economic value from tourism activities can be attributed to natural capital, is that none can then be attributed to other factors of production, such as physical or human capital, as to do so would double count the value. Therefore, for the purposes of natural capital accounting, there is a need to disaggregate the economic value between natural capital contribution and other factors of production. As a result, there will inevitably be a lower value for the natural capital contribution than would be the case from approaches which identify the total value *supported* by natural capital.

The ‘top-down’ approach utilised in this study is similar to that applied by Spalding et al. [4] for a specific activity: reef-based tourism. However, Spalding et al. [4] focused on geographic disaggregation of tourism within a country first, using geo-tagged photos and infrastructure data, before removing non-reef based expenditure using a distance based rule similar to our approach for defining activity area. Defining the T&OL activity area in which natural capital is contributing relies on broad assumptions based on distance in meters, for instance, from a footpath or fishing location. The strength of this method is that these rules are easily adjustable when further information is available and it can utilise open source data layers allowing the method to be applied to other areas. There are some tensions in the methodology where, for example, expenditure for “sightseeing/ exploring the countryside” is estimated as being 100% attributable to natural capital although it can be argued that some other capital is necessary for this activity to exist (e.g. [7]), and information on physical capital such as footpaths and roads has been used to identify where it is occurring. However, the assumption that the majority of this activity occurs on footpaths is a robust one, though in principle this activity could occur without any physical capital.

The strength of this rule-based approach is that it allows mapping of likely activity where the necessary requirements are fulfilled. However, it does not allow hotspot mapping as seen in Spalding et al. [4] as actual usage is unknown. Currently activities are assumed to occur equally across the areas identified, with all areas of a particular ecosystem attributed equal value. In reality, certain factors exist that will result in a greater or lesser use. For instance, activities may be concentrated in national parks, as the natural capital inside may be perceived as better than that outside [8], or access to marked trails easier. Like the accessibility factor utilised to spatially disaggregate spend for “visiting a beach”, for certain activities natural capital may only be utilised for an activity when other requirements are met. To incorporate actual usage, user generated content (social media) could be used to drive spatial disaggregation [4, 38]. However, using social media data comes with its own caveats [39] such as bias in user age and site popularity, which may lead to an incomplete assessment of spatial patterns of use, through missing or under-representing major groups of users who don’t tend to engage with social media. Another alternative would be to employ tools such as Social Values for Ecosystem Services (SolVES, http://solves.cr.usgs.gov) [e.g. 40–42]: which uses preference data gained through surveys to map social value index, and models the relationship between landscape metrics and social value index [43, 44]. Either approach, or a combination, could feasibly be used to direct GIS disaggregation of natural capital tourism expenditure, but is not an approach that could easily be scaled up to a national assessment.

The approach taken within this study may under-estimate the contribution of natural capital for a number of reasons. Data from national surveys may only partially account for the fuller picture of activities that are undertaken, but not reported. The economic data for valuing the natural capital contribution to T&OL expenditure is solely consumer expenditure reported in tourism surveys, and does not incorporate household capital expenditure. However, a portion of spend on T&OL capital items, such as personal equipment required for an activity, would in theory be attributable to natural capital, but is not captured in the trip-based expenditure method. Secondly, expenditure by public sectors, for instance maintenance and conservation activities funded by national and local governments, can be thought of as providing expenditure on users’ behalf so in theory could also be included. Thirdly, private businesses also spend money on physical capital to support their T&OL related commercial activities, and some of this expenditure directly leads to consumer expenditure in the form of equipment rental and entrance fees for example, or is indirectly captured, such as greater spend in restaurants with good views. As both direct and indirect commercial expenditures should be passed onto consumers through prices and fees for goods and services, this should be captured in the expenditure data, although positive externalities may occur and benefits accrue to others than the paying customer. How to include other forms of expenditure data would require further thought due to the complexity of these data types. Although using national trip-based expenditure data is not without its limitations, this method is currently the most robust for valuing natural capital contribution to T&OL. T&OL within this study was defined as trips over three hours, and excludes short duration recreational visitors who are not associated with substantial expenditure.

There exists a degree of uncertainty with activity based expenditure data that is difficult to quantify. The structure of the national surveys used as the main data source in this study led to challenges in determining the degree to which the activity motivated the visit. The relative scoring of activity ‘importance’ in motivating the visit by respondents is subjective and requires assumptions to interpret as a quantified proportion attributable to total visit expenditure. Additionally, some activity typologies were rather broad, with potential for overlaps between similarly termed activities. There is scope to improve the robustness of attributing activity-specific data, for example via surveys on a wide range of T&OL activities to better understand the motivations of visitors, and to incorporate different attributions for different types of visitors.

The alternative approach of applying the ‘bottom-up’ method would be to obtain per person per visit *activity* expenditure, the same ecosystem attribution rules would still need to be applied. With current data this is not possible, and developing such a model for each activity would be rather complex. Sen et al. [35] used socioeconomic and demographic data, travel time, availability of similar sites, visitation rates, and land cover type to predict expected visits and the per person per trip value of a recreational site. Although we have identified where the activity is likely to be occurring, generating a similar model to estimate tourism expenditure per activity would have the added challenge of different preferences of domestic and international tourists [45] and potential differences in their per activity per trip expenditure, as well as greater spatial variation in tourism activity type across the country. This method could be applied per activity to the case study area to validate the current activity based attribution in top-down approach, but would need additional surveys on per person per visit activity expenditure. A general limitation with this method is the detailed per trip per activity expenditure data required, as well as capturing the temporal change in tourism expenditure–and consequently natural capital contribution–which is possible through the current top-down approach.

## Conclusions

This study provides valuable information that underpins the importance of protecting and investing in the UK’s environmental assets. To the authors knowledge it is the first attempt to specifically identify and value the contribution of natural, separate from other, capital to T&OL activities based on available expenditure data at a national scale.

This work helps develop an approach to calculate the natural capital account for tourism at national and regional level, by taking analysis a step further from assigning full economic value of T&OL activities to natural capital, and is suitable for building natural capital national accounts in the context of tourism. For some purposes of accounting, assigning full economic value of T&OL activities may not be an issue, for example demonstrating the total value *dependent* on natural capital. But for a national accounting approach, where the purpose is to determine the natural capital contribution to T&OL, double counting with the contribution from other capitals should be avoided.

An objective was to use existing and readily available data, and although there are caveats and limitations to this approach which are discussed above, the method provides a clear starting point for how the contribution of natural capital can be extracted. Going forward, improvements in the structure of national surveys would help to disentangle further the contribution of natural capital compared to other capital. The simple, transparent methodology applied in this study allows further input of data to aid refinement of the methodology. Further improvements in the spatial disaggregation aspect could draw on individual trip-based modelling, the use of geolocated social data, and preference data, though this could lead to a more complex method that might be difficult to replicate widely.

This study fills a significant evidence gap by linking the dependency of a subset of the economic activity of the tourism sector, and thus the economy of the UK as a whole, directly to underlying ecosystem assets. This evidence can help place the environment alongside other economic considerations in decision-making by understanding its value in the same terms that economic impact is often discussed (i.e. monetary values, effect on GDP). The assessment approach can help to support the case for sustainable management of the environment to maintain the economic value of the tourism sector. In this context, a case can be made that it is in the best *economic* interest of the tourism sector to practice sustainability to help ensure a long-term viable tourism product.

## Supporting information

S1 File Worked example: Golf. (DOCX)

S2 File Full activity list. T&OL activities identified from three national surveys.
(DOCX)

S1 Table Location contribution percentages for each activity. (DOCX)

S2 Table Activity disaggregation rules applied. (DOCX)

S1 Fig Additional terrestrial activities. Location and type of ecosystems contributing to additional nine activities utilised in case study.
(DOCX)
