# Supplementary material for: Under the influence of nature: The contribution of natural capital to tourism spend
Source: PLoS One. 2022 Jun 22;17(6):e0269790. doi: 10.1371/journal.pone.0269790 (PMC9216563; doi:10.1371/journal.pone.0269790)
Supplement: S1 File — (DOCX) [file pone.0269790.s001.docx]

## Worked Example: Golf

### Calculating expenditure attributable to T&OL activities

The method for attributing expenditure to individual activities is applicable for the requirements of this study as it pertains to the common situation in which multiple activities are identified as motivating the trip. The steps are as follows:

1. Measure the total expenditure associated with trips including the activity
2. Estimate the extent to which individual activities are the reason for taking a trip
3. Identify the average number of other activities undertaken on trips
4. Use data from steps 2 and 3 (if applicable) to calculate the share of spend attributable to each activity

#### National example

This is a worked example for *played golf* across Great Britain.

*Step 1: Measure total value of trips including the activity – GBDVS, GBTS, IPS*

Total expenditure from each survey for golf is shown below.

| Total GB expenditure | GBDVS expenditure | GBTS expenditure | | IPS expenditure | |
| --- | --- | --- | --- | --- | --- |
| £ million | £ million | % of GBTS total | £ million | % of IPS total | £ million |
| £2,464 | £1,487 | 1% | £296 | 2% | £681 |

*Step 2: Estimate the extent to which the individual activities are the reason for taking the trip – GBDVS and GBTS*

Determining the extent to which an activity was the reason for taking a trip is only possible for domestic tourism. The purpose rating used here is from the OMNIBUS survey results, which provides a purpose rating profile for each activity by each trip type (i.e. holiday or short break referring to GBTS or day-trip referring to GBDVS). The purpose rating profiles for *played golf* are shown below for each survey type. Note that it is assumed that expenditure is not divided across all six purpose ratings, but rather, activity expenditure will be divided across three reasons: sole/ very important/ fairly important.

| Golf | Total unweighted based | 1- Sole reason | 2- Very important reason | 3- Fairly important reason | 4- Small reason | 5- No importance | 6- Don’t know |
| --- | --- | --- | --- | --- | --- | --- | --- |
| GBDVS | 18 | 5 | 6 | 3 | 4 | 0 | 0 |
| GBTS | 34 | 4 | 6 | 10 | 9 | 4 | 1 |

*Step 3: Identify the average number of activities undertaken on trips – GBDVS and GBTS*

The average number of activities undertaken on trips that are rated as “very important” or “fairly important” is estimated to account for people undertaking multiple activities on a single trip. The estimated average also considers non-T&OL activities (i.e. activities outside of the 24 activities) such as attending the theatre or visiting museums or art galleries. The average for *played golf* in the GBDVS is 1.0 and in GBTS is 3.2.

*Step 4: Calculating the share of spend attributable to each activity – GBDVS, GBTS and IPS*

Using the outputs from steps 2 and 3 and the measure of total trip value from Step 1, attributable expenditure for each activity can be estimated for domestic tourists. The table below shows how each steps’ output feeds into the calculation of attributable spend to each activity for domestic tourists.

|  | **GBDVS** | **GBTS** |
| --- | --- | --- |
| Total value of trips including the activity *(from Step 1) (a)* | £1,487 million | £296 million |
| % of visits where activity is the sole reason *(from Step 2) (b)* | 28% | 12% |
| % of visits where activity is very or fairly important reason *(from Step 2) (c)* | 50% | 47% |
| Average number of activities undertaken on visits that are very or fairly important *(from Step 3) (d)* | 1.0 | 3.2 |
| Share of importance of visits:  *1/ (d)* | 100% | 31% |
| Expenditure attribution factor:  *(b) + ( (c) * (d) ) = (e)* | 28% + (50% * 100%) = 78% | 12% + (47% * 31%) = 27% |
| Spend attributable to an activity:  *(e) * (a)* | £1,156 million | £79 million |

Overseas tourism takes a simpler approach to estimating attributable spend for each activity from overseas tourists, which is based on expenditure from the IPS. The table below shows how the calculation is undertaken. Based on this process, estimated attributable expenditure for *played golf* in 2017 is the sum across all three surveys, which is £1,366 million.

|  | **IPS** |
| --- | --- |
| Total value of trips including the activity *(from Step 1)* | £681 million |
| Attributable share *(based on TNS (1))* | 19% |
| Spend attributable to an activity: *Attributable share * Total value of trips* | £131 million |

#### Regional example

This example provides estimates for Wales for *played golf*. The same methodology has been applied to all regional boundaries.

*Step 1: Measure total value of trips including the activity*

Estimated total expenditure for golf in the whole of Wales are shown below.

| **Region** | **Total expenditure** | **GBDVS expenditure** | **GBTS expenditure** | |
| --- | --- | --- | --- | --- |
|  | *£ million* | *£ million* | *% of GBTS total* | *£ million* |
| Wales | £71 | £36 | 2% | £35 |

*Step 4: Calculating the share of spend attributable to each activity*

Using the measure of total trip value from Step 1, attributable expenditure for each activity can be estimated for domestic tourists at each geographical boundary. The tables below illustrate the calculation for the whole of Wales. Based on this process, estimated attributable expenditure for each activity in 2017 is the sum across both surveys, for Wales roughly £37 million.

| **Region** | **Steps** | **GBDVS** | **GBTS** | **Total** |
| --- | --- | --- | --- | --- |
| Wales | Total value of trips including the activity *(from Step 1)* | £36 million | £35 million | £71 million |
|  | Attributable share % *(from GB activity expenditure process)* | 78% | 27% |  |
|  | Spend attributable to an activity:  *Attributable share % * Total value of trips* | £28 million | £9 million | £37 million |

### Attributing portion of activity expenditure to natural capital

The location contribution and ecosystem contribution for played golf following breakdown of visits from GBDVS and answering framing questions is shown below:

| **Location category** | **Location contribution** | **Would the activity occur without the ecosystems?** | **Would the activity occur without other capital?** | **Ecosystem Contribution** | **Activity is….** |
| --- | --- | --- | --- | --- | --- |
| Built-up | 16% | Partial | No | 25% | Nature-incidental (Nature secondary to experience) |
| Rural | 42% | No | No | 50% | Nature-enhanced  (Nature moderately improves experience) |
| Coastal | 44% | No | No | 50% | Nature-enhanced (Nature moderately improves experience) |

The final ecosystem attribution for *played golf* is 47% (sum product of location contribution multiplied by ecosystem contribution), equating to approximately £17 million for the whole of Wales.

### Spatial allocation case study for Pembrokeshire

Based on the three year weighted average of 2015-2017 GBDVS data, Pembrokeshire is approximately to 5.01% of national value for wales. This is applied to all activities, therefore, for *played golf* this equates to approximately £874,637.

Activity specific GIS rules to generate boundary polygon within which natural capital is assumed to be contributing to *played golf* is as follows:

| **Activity** | **Data** | **Pre-processing of layers** | **Spatial disaggregation** | **Assumptions** |
| --- | --- | --- | --- | --- |
| Played Golf | OS Open Greenspace^[[1]](#footnote-1)^  LCM2015^[[2]](#footnote-2)^  Marine Management Organisation Land with Sea Views^[[3]](#footnote-3)^ | Selected greenspace with Function = “golf course” | Applied a 500m buffer to golf courses. Extracted habitat from LCM2015 within this 500m buffer. Extracted area with sea views within this 500m buffer. | Assume visits are influenced by location, and therefore habitat, surrounding the golf course. |

Resulting boundary polygons for *played golf* is shown below. Marine is for illustration only, Land with Sea Views [2] was used to calculated contribution of this ecosystem type.


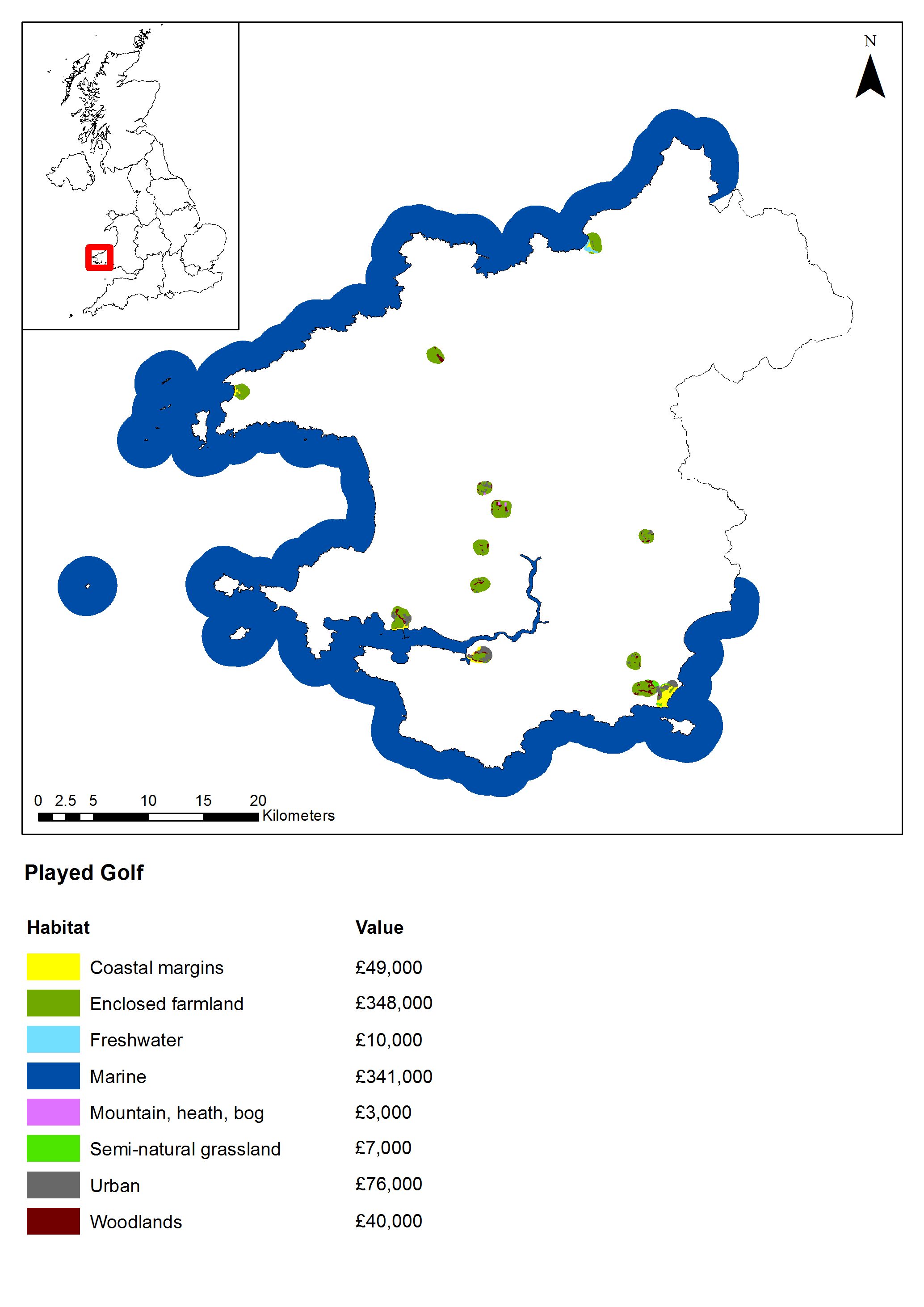

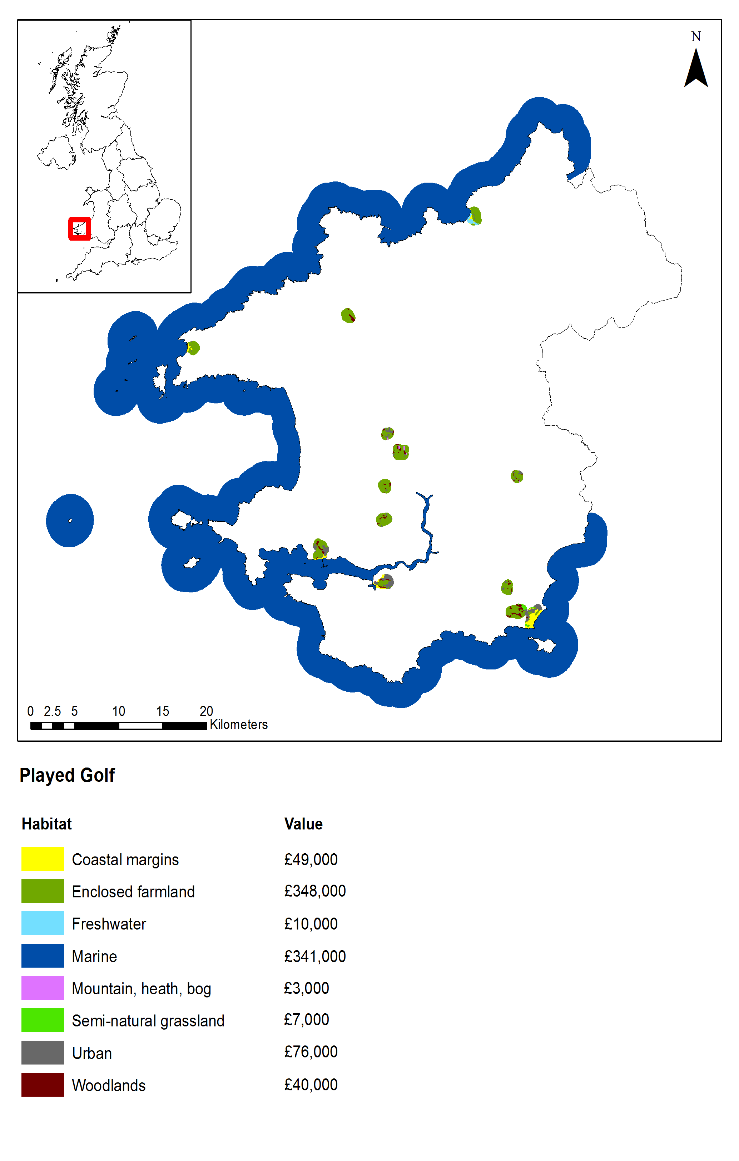


Area of ecosystem type within the boundary polygons was then extracted, the resulting proportion and attribution of expenditure based on this for *played golf* is shown below. Note that golf courses on or near the coastal margin habitat of sand dunes are assumed to be links courses and therefore accrue a higher value.

| **Ecosystem type** | **Percentage of ecosystem type within boundary** | **Habitat weighting for that activity** | **Total expenditure attribution to ecosystem type** |
| --- | --- | --- | --- |
| Coastal margins | 6% | 2.0 | £ 92,000 |
| Enclosed farmland | 40% | 1.0 | £ 330,000 |
| Freshwater | 1% | 1.0 | £ 10,000 |
| Marine by proxy | 39% | 1.0 | £ 323,000 |
| Mountain, heath, bog | 0% | 1.0 | £ 3,000 |
| Semi-natural grassland | 1% | 1.0 | £ 7,000 |
| Urban | 9% | 1.0 | £ 72,000 |
| Woodlands | 5% | 1.0 | £ 38,000 |

## References

1. TNS. Valuing Activities. 2015. Available from: https://www.visitbritain.org/sites/default/files/vb-corporate/Documents-Library/documents/England-documents/valuing_activities_-_final_report_fv_7th_october_2015_0.pdf

2. MarineManagementOrganisation. Land with Sea Views. In: MarineManagementOrganisation, editor.: Government Digital Service 2016.

1. Contains OS data © Crown copyright and database right (2019) [↑](#footnote-ref-1)
2. Rowland, C.S.; Morton, R.D.; Carrasco, L.; McShane, G.; O'Neil, A.W.; Wood, C.M. (2017). Land Cover Map 2015 (vector, GB). NERC Environmental Information Data Centre. <https://doi.org/10.5285/6c6c9203-7333-4d96-88ab-78925e7a4e73> [↑](#footnote-ref-2)
3. © Crown copyright [↑](#footnote-ref-3)
