# Supplementary material for: Under the influence of nature: The contribution of natural capital to tourism spend
Source: PLoS One. 2022 Jun 22;17(6):e0269790. doi: 10.1371/journal.pone.0269790 (PMC9216563; doi:10.1371/journal.pone.0269790)
Supplement: S2 File — T&OL activities identified from three national surveys. (DOCX) [file pone.0269790.s002.docx]

## T&OL activities identified from three national surveys

The additional 8 activities identified through the IPS detailed activity list [1] are shown in bold. Activities in *italics* were those not undertaken for the case study area

- ***Adventure sports (e.g. skiing, snowboarding, rafting, canyoning)***
- Cycling or mountain biking
- Eating and drinking locally produced food and drink
- Fishing – sea angling, coarse fishing, game fishing
- Going to visitor attractions such as theme parks, gardens, famous buildings, museums, zoos etc.
- ***Had a picnic or BBQ***
- ***Horse riding, pony trekking***
- Long walks, hikes or rambles (minimum of 2 miles/ 1 hour)
- Played golf
- ***Running, jogging, orienteering***
- ***Short walk/stroll (up to 2 miles/ 1 hour)***
- Sightseeing / exploring at the coast
- Sightseeing / exploring at the countryside
- *Sightseeing in a town or city*
- ***Sightseeing on foot***
- ***Sunbathing***
- **Visited a beach**
- Visited a location associated with a TV series, film or literature
- Visiting a cathedral, church, abbey or other religious building
- Visiting historic buildings or monuments (e.g. castles, stately homes)
- Visiting parks or gardens
- Visiting zoos, aquariums or wildlife attractions
- Watching wildlife, bird watching, other nature
- Watersports including sailing, canoeing, kayaking, windsurfing, etc.

## References

1. TNS. Valuing Activities. 2015. Available from: https://www.visitbritain.org/sites/default/files/vb-corporate/Documents-Library/documents/England-documents/valuing_activities_-_final_report_fv_7th_october_2015_0.pdf
