# Supplementary material for: Under the influence of nature: The contribution of natural capital to tourism spend
Source: PLoS One. 2022 Jun 22;17(6):e0269790. doi: 10.1371/journal.pone.0269790 (PMC9216563; doi:10.1371/journal.pone.0269790)
Supplement: S1 Table — (DOCX) [file pone.0269790.s003.docx]

**Location contribution percentages for each activity**

| **Activity** | **Built up** | **Rural** | **Coastal** |
| --- | --- | --- | --- |
| Adventure sports (e.g. skiing, snowboarding, rafting, canyoning) | 6% | 21% | 73% |
| Cycling or mountain biking | 10% | 23% | 67% |
| Eating and drinking locally produced food and drink | 14% | 30% | 56% |
| Fishing – sea angling, coarse fishing, game fishing | 13% | 43% | 44% |
| Going to visitor attractions such as theme parks, gardens, famous buildings, museums, zoos etc. | 13% | 26% | 61% |
| Had a picnic or BBQ | 10% | 32% | 58% |
| Horse riding, pony trekking | 18% | 39% | 43% |
| Long walks, hikes or rambles (minimum of 2 miles/ 1 hour) | 14% | 34% | 52% |
| Played golf | 12% | 31% | 57% |
| Running, jogging, orienteering | 8% | 21% | 71% |
| Short walk/stroll (up to 2 miles/ 1 hour) | 16% | 29% | 54% |
| Sightseeing / exploring at the coast | 0% | 0% | 100% |
| Sightseeing / exploring at the countryside | 0% | 100% | 0% |
| Sightseeing in a town or city | 100% | 0% | 67% |
| Sightseeing on foot | 11% | 23% | 67% |
| Sunbathing | 13% | 25% | 63% |
| Visited a beach | 4% | 10% | 86% |
| Visited a location associated with a TV series, film or literature | 16% | 32% | 52% |
| Visiting a cathedral, church, abbey or other religious building | 10% | 23% | 67% |
| Visiting historic buildings or monuments (e.g. castles, stately homes) | 12% | 26% | 62% |
| Visiting parks or gardens | 11% | 35% | 55% |
| Visiting zoos, aquariums or wildlife attractions | 9% | 22% | 69% |
| Watching wildlife, bird watching, other nature | 6% | 25% | 69% |
| Watersports including sailing, canoeing, kayaking, windsurfing, etc | 14% | 19% | 67% |
