# Supplementary material for: Under the influence of nature: The contribution of natural capital to tourism spend
Source: PLoS One. 2022 Jun 22;17(6):e0269790. doi: 10.1371/journal.pone.0269790 (PMC9216563; doi:10.1371/journal.pone.0269790)
Supplement: S2 Table — (DOCX) [file pone.0269790.s004.docx]

## Activity disaggregation rules and data sources

| Activity | Data | Pre-processing of layers * | Spatial disaggregation | Assumptions | Caveats and further refinement |
| --- | --- | --- | --- | --- | --- |
| Played Golf | OS Open Greenspace^[[1]](#footnote-1)^  LCM2015^[[2]](#footnote-2)^  Marine Management Organisation Land with Sea Views^[[3]](#footnote-3)^ | Selected greenspace with Function = “golf course” | Applied a 500m buffer to golf courses. Extracted habitat from LCM2015 within this 500m buffer. Extracted area with sea views within this 500m buffer. | Assume visits are influenced by location, and therefore habitat, surrounding the golf course. |  |
| Long walks, hikes, or rambles (minimum of 2 miles/1hour) | Active Travel Approved Routes^3^  National Trails^[[4]](#footnote-4)^  Wales Coast Path^4^  NRW Forest Recreation Routes^4^  NRW Forest Roads^4^;  Open Access Open country^4^  Open Access Registered Common Land^4^  National Parks^4^  ORVal Paths^[[5]](#footnote-5)^  LCM2015^2^  Marine Management Organisation Land with Sea Views^3^ | Selected active travel routes with PathDesign of: 'tf_foot' , 'tf_shared', 'tf_shared_away', and 'tf_segregated' | Applied a 500m buffer to linear networks. Extracted habitat from LCM2015 within this 500m buffer. Extracted area with sea views within this 500m buffer.  Mapped Open Access Open country, Open Access Registered Common Land, National Parks. Extracted habitat from LCM2015 located within these areas. Extracted area with sea views located within these areas. | That the active travel routes selected are the only active travel routes used for walking.  That countryside right of way areas and national parks are criss-crossed with trails, therefore all habitat within them is contributing.  When a footpath/active travel route enters a built-up area, the buffer will capture this urban environment and therefore account for walking in built-up areas. | Not all footpaths within Pembrokeshire have been captured, which can be seen in the disjointed nature of disaggregation.  Further refinement of this method would be to investigate a more detailed footpath layer. |
| Fishing – coarse fishing, game fishing | Angling Trust^[[6]](#footnote-6)^ | Selected Water Types of River, Stillwater, Canal  Point locations mapped | Applied a 500m buffer to point locations. Extracted habitat from LCM2015 within this 500m buffer. Extracted area with sea views within this 500m buffer. | That the Angling Trust has identified the most used freshwater fishing sites and that people are more likely to fish at sites detailed by the Angling Trust.  Assume visits are influenced by location, and therefore habitat, surrounding the fishing location. | These are point locations when in reality they refer to river stretches, known as beats. This could result in an under-representation of the area. Additionally, small lakes and ponds may not be picked up within the LCM2015 25m dataset, which could explain the lower percentage for freshwater habitat classification.  However, realistically to take this method nationally – point locations are the other dataset that will be available, and this method does allow spatial disaggregation.  Currently no spatial data layer exists, spatial data would need to be obtained through contacting the Angling Trust or NRW/EA/SEPA/DEFRA as a licence must be applied for in order to fish in freshwater. |
| Cycling or Mountainbiking  Three different forms of cycling:  • road cycling  • mountain biking (2 types: at trail centres (assume 80% of this activity happens here), & free-form (20% of activity)  • recreational family cycling  Each require different mixes of infrastructure and habitat reliance | NRW Forest Recreation Routes^4^  NRW Forest Roads^4^;  ORVal Paths^5^  Wales Coast Path^4^  Active Travel Approved^3^  OS Open Roads^1^  LCM2015^2^  Marine Management Organisation Land with Sea Views^3^ | Selected active travel routes with PathDescrip of: 'Cycle lane (on road, segregated)' , 'Cycle route (on road, not segregated)' , 'Cycle track (away from road)' , 'Cycle track (alongside a road)' , 'Segregated footpath/cycle track (alongside road)' , 'Shared use footpath/cycle track (alongside road)' , 'Shared use footpath/cycle track (away from road)'  Selected ORVal Paths that do not intersect with the Wales Coast Path.  Selected roads with function : 'Minor Road' OR 'B Road' and from these deleted anything with "form of way" that was not a single carriageway | Applied a 500m buffer to linear networks. Extracted habitat from LCM2015 within this 500m buffer. Extracted area with sea views within this 500m buffer. | That the active travel routes selected are the only active travel routes used for cycling.  As cycling is not allowed on the coastal path, that it does not occur there.  That paths identified by ORVal are suitable for cycling  That minor roads and B roads are the main type of roads used for cycling.  National parks and countryside right of way areas have not been used as a “proxy” for cycling, unlike walking, as assuming that main pathways through these areas have been picked up by path layers. | Footpaths from ORVal have been used in this methodology and though the assumption applied is that all are suitable for cycling, this is most likely a simplification.  Obtaining a GIS layer of cycle routes would improve the spatial disaggregation or this activity. Additionally, incorporation of elevation could be used to identify free-form mountain biking and constrain paths used from ORVal data. |
| Visiting historic buildings or monuments (e.g. castles, stately homes) | Enthusiast websites for castles^[[7]](#footnote-7)^ and lighthouses^[[8]](#footnote-8)^  LCM2015^2^  Marine Management Organisation Land with Sea Views^3^ | Area of Pembrokeshire selected  Point locations mapped | Applied a 500m buffer to point locations. Extracted habitat from LCM2015 within this 500m buffer. Extracted area with sea views within this 500m buffer. | That by text of “e.g. castles, stately home” historic buildings and monuments are considered fairly substantial i.e. not a small standing stones, and they are publicly accessible i.e. not a listed house  That these sites hold accurate information on castles and lighthouses within Pembrokeshire.  That most visited historic buildings and monuments within Pembrokeshire are castles and lighthouses | The results are biased towards castles and lighthouses.  There exists a Scheduled Monuments layer by CADW, however, this incorporates small monuments such as areas where prehistoric settlements have been so have not been used for this category base on first assumption.  There also exists a Listed Buildings layer by CADW, however, this contains every listed building with many that are not publicly accessible or would be visited. Through not using this layer, applicable buildings – particularly stately homes – have been missed; however, this would require sifting through the dataset manually to extract relevant, publicly accessible buildings. A suggested future refinement of the method.  Further refinement is needed to nationalise this method; data would need to be obtained regarding the locations of castles and lighthouses along with potentially other specific historic buildings, as currently no spatial data layer exists. This information may be obtainable from Historic England / Historic Wales / Historic Environment Scotland / NI Direct or VisitWales / VisitEngland / VisitScotland / Discover Northern Ireland |
| Visiting a cathedral, church, abbey or other religious building | Listed Buildings^3^  LCM2015^2^  Marine Management Organisation Land with Sea Views^3^ | Selected listed buildings with BroadClass type of : 'Religious, Ritual and Funerary' | Applied a 500m buffer to point locations. Extracted habitat from LCM2015 within this 500m buffer. Extracted area with sea views within this 500m buffer. | That religious buildings being visited will have been classed as a “*Building of Special Architectural or Historic Interest”*  That religious structures are within 500m of a religious building. | Under the class of 'Religious, Ritual and Funerary' religious structures as well as buildings will have been incorporated, however, the assumption that where there is a religious structure a religious building is nearby can apply.  Further refinement could be achieved by sifting further through this data. |
| Going to visitor attractions such as theme parks, gardens, famous buildings, museums, zoos etc. | VisitWales ^[[9]](#footnote-9)^  Internet search for museums  Enthusiast websites for castles^7^ and lighthouses^8^  OS Open Greenspace^1^  ORVal Parks^5^  LCM2015^2^  Marine Management Organisation Land with Sea Views^3^ | Theme parks searched for in area of Pembrokeshire.  Museums searched for in area of Pembrokeshire.  Points mapped | Applied a 500m buffer to point locations. Extracted habitat from LCM2015 within this 500m buffer. Extracted area with sea views within this 500m buffer. | That all locations within layer are considered a visitor attraction. | Currently this category incorporates spatially the same data as other categories: visiting historic buildings or monuments (e.g. castles, stately homes); visiting parks or gardens; and visiting zoos, aquariums or wildlife attractions. This was deemed appropriate for this study. To refine this, further information would be needed to separate out the categories.  Further refinement in the case of parks and gardens for this category could be achieved by selecting based upon size.  Currently there is no spatial data layer for visitor attractions such as theme parks, museums and art galleries. Data may be obtainable to create a layer from VisitWales / VisitEngland / VisitScotland / Discover Northern Ireland. |
| Visited a location associated with a TV series, film or literature | Wales travel website^[[10]](#footnote-10)^  Beaches_Pembrokeshire^[[11]](#footnote-11)^  LCM2015^2^  Marine Management Organisation Land with Sea Views^3^ | Locations searched for in area of Pembrokeshire.  Applicable beaches extracted. | Applied a 500m buffer to beaches. Extracted habitat from LCM2015 within this 500m buffer.  Extracted area with sea views within this buffer. | That the full beach is applicable, rather than part. | This layer is biased towards filming locations, which in Pembrokeshire are specific beaches. There are potentially locations associated with literature and TV that have been missed.  Currently no spatial data layer exists, to expand this method nationally this would be have to be created. Enthusiast websites exists which map out locations who could be contacted. |
| Sightseeing / exploring at the coast | Local Authorities^3^  LCM2015^2^  Marine Management Organisation Land with Sea Views^3^ |  | Applied a 500m buffer to coastline and extracted habitat from LCM2015 within this 500m buffer. Extracted area with sea views within this buffer. | That entire coastline is used in some form for sightseeing / exploring at the coast due to presence of coastal path.  That this activity is not constrained by vehicle access  That 500m within the coast is considered as exploring at the coast. | A potential further refinement if up-scaled in addition to identifying presence of coastal paths would be to apply a distance function from coastal population centres.    The buffer of 500m may be an overestimate for some coastal features like cliffs, but an underestimate for large dune systems. Further refinement of the method could incorporate coastal features to generate a variable sized coastal buffer. |
| Sightseeing / exploring the countryside | Scheduled Monuments^3^  The Registered Landscapes of Outstanding and of Special Interest in Wales^3^  Active Travel Approved Routes^3^  National Trails^4^  NRW Forest Recreation Routes^4^  NRW Forest Roads^4^;  Open Access Open country^4^  Open Access Registered Common Land^4^  National Parks^4^  ORVal Paths^5^  LCM2015^2^  Marine Management Organisation Land with Sea Views^3^ | Selected active travel routes with PathDesign of: 'tf_foot' , 'tf_shared', 'tf_shared_away', and 'tf_segregated' | Applied a 500m buffer to linear networks and Scheduled Monument polygons. Extracted habitat from LCM2015 within this 500m buffer. Extracted area with sea views within this 500m buffer.  Mapped Open Access Open country, Open Access Registered Common Land, National Parks, Registered Landscapes of Outstanding Special Interest in Wales. Extracted habitat from LCM2015 located within these areas. Extracted area with sea views located within these areas.  Erased output within 500m of coastline. | That the presence of landscapes of outstanding and special interest, access land, national parks and historic monuments are driving exploration of the countryside.  That exploration is being done on foot.  That 500m within the coast is considered as exploring at the coast. | Further refinement of this method would be to investigate a more detailed footpath layer, and consider other factors that would drive exploring the countryside, for example car parks away from rural centres. |
| Watching wildlife, bird watching, other nature | National Parks^4^  RSPB reserves^[[12]](#footnote-12)^  ORVal Parks^5^  Local Nature Reserves^4^  LCM2015^2^  Marine Management Organisation Land with Sea Views^3^ | Selected ORVal parks with TYPE: 'country_park' , 'nature', 'FC_woods' , 'wood' | Applied a 500m buffer to point locations of RSPB reserves. Extracted habitat from LCM2015 located within these areas. Extracted area with sea views located within these areas.  Mapped National Parks, Local Nature Reserves, ORVal Parks. Extracted habitat from LCM2015 located within these areas. Extracted area with sea views located within these areas. | That “park” category from ORVal Parks layer is more recreation not wildlife driven and therefore not applicable.  National parks, country parks, and local nature reserves have better quality habitat to support wildlife, consequently there a greater chance of seeing wildlife. Additionally, that people are visiting these locations because of this assumption. | Woodland is included, though access to all woodland patches is debateable and would need further refinement, such as incorporating an access point.  Method of attributing marine component based on area of land with sea views might not be directly applicable in this case. However, it does account indirectly for the marine habitat that many bird species will be reliant upon.  Along the coastline there will be marine mammal watching, as well as boat trips. Further refinement could incorporate a better methodology to account for the marine and coastal component |
| Visiting parks or gardens | OS Open Greenspace^1^  ORVal Parks^5^  LCM2015^2^  Marine Management Organisation Land with Sea Views^3^ | Selected greenspace with Function: 'Public Park Or Garden' Selected ORVal parks with TYPE: 'country_park' , 'park' | Mapped polygons. Extracted habitat from LCM2015 located within these areas. Extracted area with sea views located within these areas. | Assume visits are driven by the habitat inside the park and garden, opposed to the setting (like golf courses). | Small, local parks are included in the output. Further refinement of the methodology could be to constrain by park size.  Additionally, the assumption of visits driven by habitat inside the park might be deemed incorrect. If thought the wider setting should be incorporate then same methodology as that applied to golf course would be implemented. |
| Visiting zoos, aquariums or wildlife attractions | VisitWales^9^  LCM2015^2^  Marine Management Organisation Land with Sea Views^3^ | Zoos, aquariums and wildlife attractions searched for in Pembrokeshire.  Points mapped. | Applied a 500m buffer to point locations. Extracted habitat from LCM2015 located within these areas. Extracted area with sea views located within these areas. | That locations from VisitWales are all wildlife attractions in Pembrokeshire, or at least the most visited. | Currently no spatial data layer exists. Data may be obtainable to create a layer from VisitWales / VisitEngland / VisitScotland / Discover Northern Ireland. |
| Eating and drinking locally produced food and drink | CEH urban extent layer  LCM2015^2^  Marine Management Organisation Land with Sea Views^3^ |  | Applied a 2000m buffer around urban extent layer.  Extracted habitat from LCM2015 located within these areas. Extracted area with sea views located within these areas. | That local is defined as 2000m away from urban area.  Urban area contributes to locally produced food and drink. | Further refinement of the method would be to re-define the area associated with “local”.  Method of attributing marine component based on area of land with sea views is not applicable for this activity. Though it does allow attribution to the marine environment for this category, further refinement would be to create a marine buffer extending out from coastal urban areas. |
| Visiting a beach | Beaches_Pembrokeshire^12^  Open Data ESRI base maps OS_Open_Carto and World_Imagery^[[13]](#footnote-13)^  PembrokshireCoast^[[14]](#footnote-14)^ | Locations of car parks within Pembrokeshire searched for  Point locations mapped | Applied a 100m buffer around car park points. Extracted beach polygons that intersect with the 100m car park buffer and assigned field “Y”. Beach polygons that do not intersect with car park buffer assigned field “N”. | It is assumed that beaches with good access (i.e. roads and car parks) are likely to receive higher proportion of visitors regardless of beach type (e.g sand, shingle, rock or size.  Since car parks can be used as indicators of road access, there was no need to map the road network. | A potential refinement is to incorporate beach type and size, as well as car park size. The percentage of expenditure was assigned evenly to each beach category regardless of the size (area) of the beach or underlying habitat as they were not defined in this data layer.  Identifying beaches with no car parks nearby was undertaken manually. An additional buffer measure could be implemented to refine process. |
| Fishing - sea angling  Category split into boat-based angling and short angling | OS Mean Low Water (MLW)^2^  OS Foreshore^2^  UKHO 6 nautical mile limit^[[15]](#footnote-15)^ |  | ‘Marine area’ mapped based on 6 nautical mile limit. | Boat-based angling was assumed to take place from a boat between the mean low water limit to 6 nautical miles offshore. No sea angling was assumed to take place further than 6 nautical miles offshore.  Shore angling was assumed to take place in the intertidal/foreshore area. | There is no central source of information regarding where angling occurs, hence lack of spatial disaggregation.  Improvements would be to obtain spatial data from contacting sea angling/boat charter organisations of possible from NRW/EA/SPEA/Defra |
| Watersports  Category split into boardsports and vessel-based watersports | OS Mean Low Water (MLW)^2^  UKHO 6 nautical mile limit^16^ |  | Applied 500m buffer from MLW to create ‘near-shore waters’  ‘Marine area’ mapped based on 6 nautical mile limit and minus ‘near-shore waters’ | Assumed all boardsports occur 500m from the coast, and vessel based watersports occur greater than 500m from coast but less than 6 nautical miles.  Watersports are not defined in the source data, therefore, it was assumed to include the following activities:  Boardsports (e.g. surfing, kitesurfing, wind surfing, paddle-boarding)  Vessel/water craft based watersports (e.g. kayaking, canoeing, dinghy sailing, yachting, personal water craft)  Sub-activities that were not included were scuba diving (assumed to be in Adventure Sports category) and sea swimming (assumed included in visiting the beach). | Improvements would be to clarify activities assumed to be watersports, and to potentially restrain layers using beach type, access points, watersports gear rental locations, and locations for specific watersports activities based on conditions i.e. sheltered coves. |

| Data layer | File Name | Link or Hold location |
| --- | --- | --- |
| OS Open Greenspace^1^ | opgrsp_gml3_sm  opgrsp_gml3_sn | <https://www.ordnancesurvey.co.uk/opendatadownload/products.html#OPGRSP> |
| LCM2015^2^ | lcm2015_gb_1km_dominant_aggregate_class | GB: <https://catalogue.ceh.ac.uk/documents/711c8dc1-0f4e-42ad-a703-8b5d19c92247> |
| Marine Management Organisation Land with Sea Views^3^ | Sea_Visibility_from_Land.shp | <https://data.gov.uk/dataset/241acfd9-774f-4d8b-b7c4-cb4b8c324657/marine-management-organisation-land-with-sea-views> |
| Active Travel Approved Routes^3^ | activetravel_routesection_approvedroutes.shp | <http://lle.gov.wales/catalogue/item/ActiveTravelApprovedRoutes/?lang=en> |
| National Trails^4^ | National Trails | <http://lle.gov.wales/catalogue/item/NationalTrails/?lang=en> |
| Wales Coast Path^4^ | Wales Coast Path | <http://lle.gov.wales/catalogue/item/WalesCoastalPath/?lang=en> |
| Open Access Open country^4^ | NRW_OPEN_COUNTRY_2014Polygon.shp | <http://lle.gov.wales/catalogue/item/OpenAccessOpenCountry/?lang=en> |
| Open Access Registered  Common Land^4^ | NRW_COMMON_LAND_2014Polygon.shp | <http://lle.gov.wales/catalogue/item/OpenAccessRegisteredCommonLand/?lang=en> |
| National Parks^4^ | NRW_NATIONAL_PARKPolygon.shp | <http://lle.gov.wales/catalogue/item/NationalParks/?lang=en> |
| NRW Forest Recreation Routes^4^ | NRW_GB_RECREATION_ROUTESLine.shp | <http://lle.gov.wales/catalogue/item/NationalForestEstateRecreationRoutes/?lang=en> |
| NRW Forest Roads^4^ | NRW_FOREST_ROADSLine.shp | <http://lle.gov.wales/catalogue/item/NationalForestEstateRoads/?lang=en> |
| ORVal Paths^5^ | paths_wales.shp | <https://www.leep.exeter.ac.uk/orval/documents> |
| OS Open Roads^1^ | oproad_essh_gb | <https://www.ordnancesurvey.co.uk/opendatadownload/products.html#OPROAD> |
| Listed Buildings^3^ | Cadw_ListedBuildingsPoint.shp | <http://lle.gov.wales/catalogue/item/ListedBuildings/?lang=en> |
| ORVal Parks^5^ | parks_wales.shp | <https://www.leep.exeter.ac.uk/orval/documents> |
| Beaches_Pembrokeshire^10^ | Beaches_Pembrokeshire | ABPmer |
| Local Authorities^3^ | localauthorities_lwm.shp | <http://lle.gov.wales/catalogue/item/LocalAuthorities/?lang=en> |
| Scheduled Monuments^3^ | Cadw_SAMPolygon.shp | <http://lle.gov.wales/catalogue/item/ScheduledAncientMonumentsInWales/?lang=en> |
| The Registered Landscapes of Outstanding and of Special Interest in Wales^3^ | Cadw_HistoricLandscapesPolygon.shp | <http://lle.gov.wales/catalogue/item/RegisteredLandscapesOfOutstandingHistoricInterestInWales/?lang=en> |
| RSPB reserves^11^ | RSPB_reserves.kmz | <https://www.rspb.org.uk/reserves-and-events/reserves-a-z/rspb-reserves-on-google-earth/> |
| Local Nature Reserves^4^ | NRW_LNRPolygon.shp | <http://lle.gov.wales/catalogue/item/LocalNatureReserves/?lang=en> |
| CEH urban extent layer | UrbanExtent.shp | CEH |

1. Contains OS data © Crown copyright and database right (2019) [↑](#footnote-ref-1)
2. Rowland, C.S.; Morton, R.D.; Carrasco, L.; McShane, G.; O'Neil, A.W.; Wood, C.M. (2017). Land Cover Map 2015 (vector, GB). NERC Environmental Information Data Centre. <https://doi.org/10.5285/6c6c9203-7333-4d96-88ab-78925e7a4e73> [↑](#footnote-ref-2)
3. © Crown copyright [↑](#footnote-ref-3)
4. Contains Natural Resources Wales information © Natural Resources Wales and Database Right. All rights Reserved. Contains Ordnance Survey Data. Ordnance Survey Licence number 100019741. Crown Copyright and Database Right. [↑](#footnote-ref-4)
5. Day, B. H., and G. Smith (2018). Outdoor Recreation Valuation (ORVal) User Guide: Version 2.0, Land, Environment, Economics and Policy (LEEP) Institute, Business School, University of Exeter. [↑](#footnote-ref-5)
6. [https://fishinginfo.co.uk/index.html#searchresults](https://fishinginfo.co.uk/index.html%23searchresults%20) [↑](#footnote-ref-6)
7. <https://www.historic-uk.com/HistoryMagazine/DestinationsUK/CastlesinWales/> [↑](#footnote-ref-7)
8. <http://www.lighthousesrus.org/Maps/mapSql.php?page=UK/Wales> [↑](#footnote-ref-8)
9. <https://www.visitwales.com/search?search=Wales&f%5B0%5D=sector%3A5001> [↑](#footnote-ref-9)
10. <https://www.holidaycottages.co.uk/blog/top-10-films-and-tv-series-filmed-in-wales> [↑](#footnote-ref-10)
11. Dewey, N. and Roberts, C. ABPmer [↑](#footnote-ref-11)
12. ©The Royal Society for the Protection of Birds [↑](#footnote-ref-12)
13. World imagery basemap: Esri, DigitalGlobe, GeoEye, Earthstar Geographics, CNES/Airbus DS, USDA, USGS, AeroGRID, IGN, and the GIS User Community [↑](#footnote-ref-13)
14. <https://www.pembrokeshirecoast.wales/> [↑](#footnote-ref-14)
15. UKHO © Crown copyright [↑](#footnote-ref-15)
