# Supplementary material for: Under the influence of nature: The contribution of natural capital to tourism spend
Source: PLoS One. 2022 Jun 22;17(6):e0269790. doi: 10.1371/journal.pone.0269790 (PMC9216563; doi:10.1371/journal.pone.0269790)
Supplement: S1 Fig — Location and type of ecosystems contributing to additional nine activities utilised in case study. (DOCX) [file pone.0269790.s005.docx]

## Location and type of ecosystems contributing to additional nine activities utilised in

## **
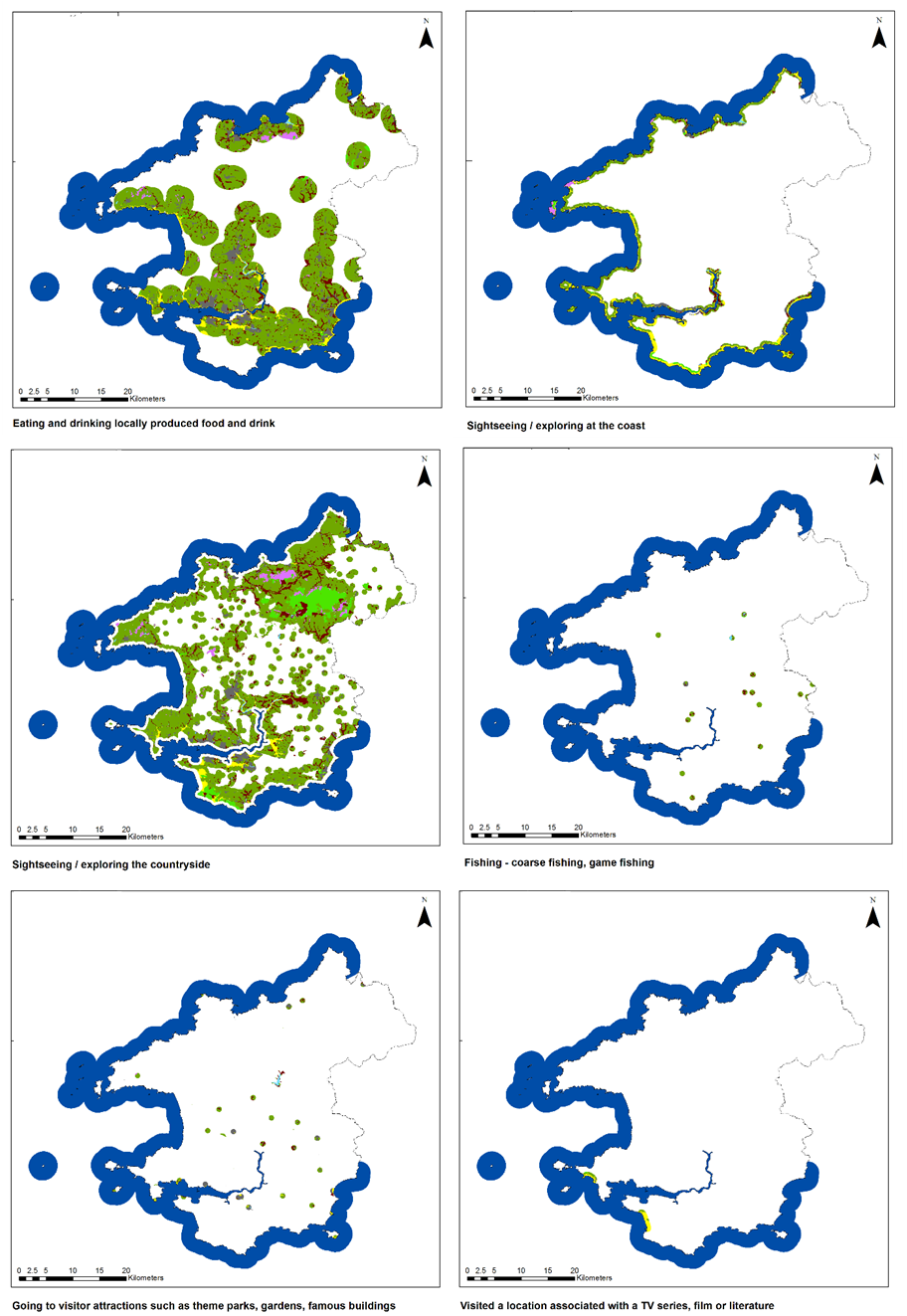
**case study.

**
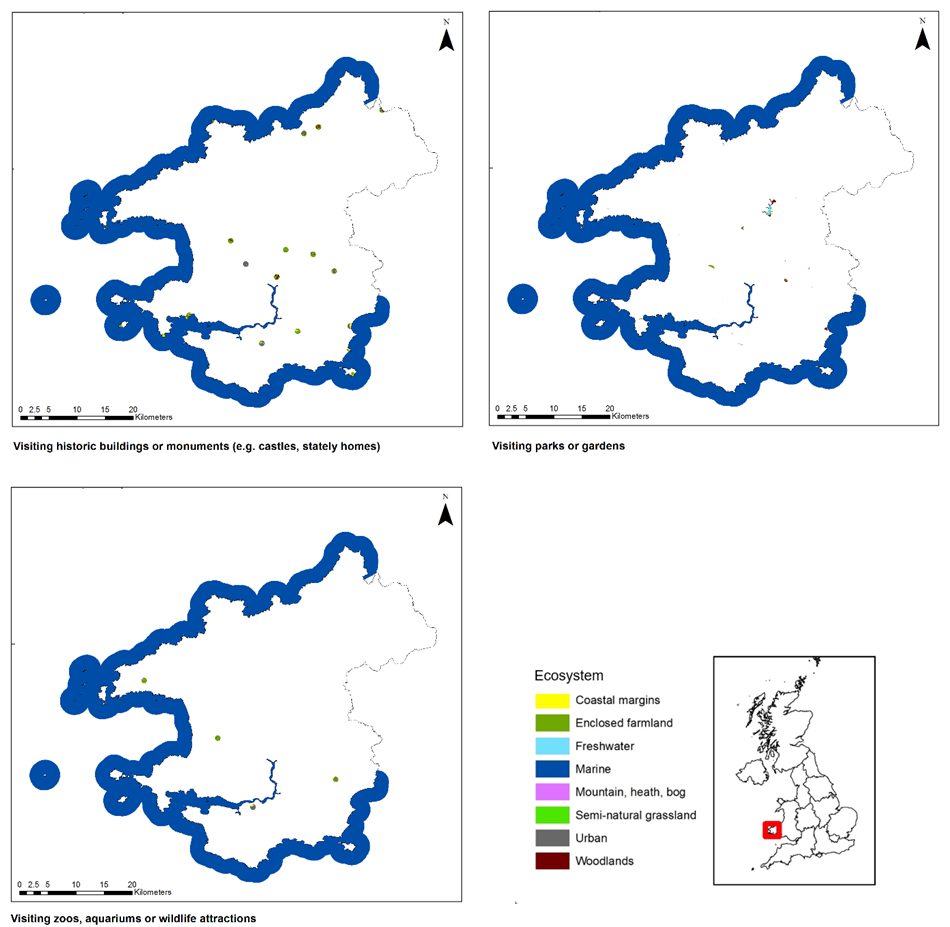
**

*Includes boundary of Pembrokeshire and NUTS1 regions. Source: Office for National Statistics licenced under the Open Government Licence v3.0. Contains OS data © Crown copyright and database right 2019*
